# Discovery of BRAF/HDAC Dual Inhibitors Suppressing Proliferation of Human Colorectal Cancer Cells

**DOI:** 10.3389/fchem.2022.910353

**Published:** 2022-07-22

**Authors:** Yingjun Li, Yongjun Huang, Huimin Cheng, Fang Xu, Ruxi Qi, Botao Dai, Yujian Yang, Zhengchao Tu, Lijie Peng, Zhang Zhang

**Affiliations:** ^1^ Academy for Advanced Interdisciplinary Studies and Department of Chemistry, Southern University of Science and Technology, Shenzhen, China; ^2^ International Cooperative Laboratory of Traditional Chinese Medicine Modernization and Innovative Drug Development of Chinese Ministry of Education (MOE), Guangzhou City Key Laboratory of Precision Chemical Drug Development, School of Pharmacy, Jinan University, Guangzhou, China; ^3^ XtalPi Inc., (Shenzhen Jingtai Technology Co., Ltd.), Shenzhen, China; ^4^ Cryo-EM Center, Southern University of Science and Technology, Shenzhen, China; ^5^ Guangzhou Institutes of Biomedicine and Health, Chinese Academy of Sciences, Guangzhou, China

**Keywords:** BRAF, histone deacetylase, dual inhibitor, colorectal cancer, HDAC

## Abstract

The combination of histone deacetylase inhibitor and BRAF inhibitor (BRAFi) has been shown to enhance the antineoplastic effect and reduce the progress of BRAFi resistance. In this study, a series of (thiazol-5-yl)pyrimidin-2-yl)amino)-*N*-hydroxyalkanamide derivatives were designed and synthesized as novel dual inhibitors of BRAF and HDACs using a pharmacophore hybrid strategy. In particular, compound **14b** possessed potent activities against BRAF, HDAC1, and HDAC6 enzymes. It potently suppressed the proliferation of HT-29 cells harboring BRAF^V600E^ mutation as well as HCT116 cells with wild-type BRAF. The dual inhibition against BRAF and HDAC downstream proteins was validated in both cells. Collectively, the results support **14b** as a promising lead molecule for further development and a useful tool for studying the effects of BRAF/HDAC dual inhibitors.

## 1 Introduction

**FIGURE 1:**
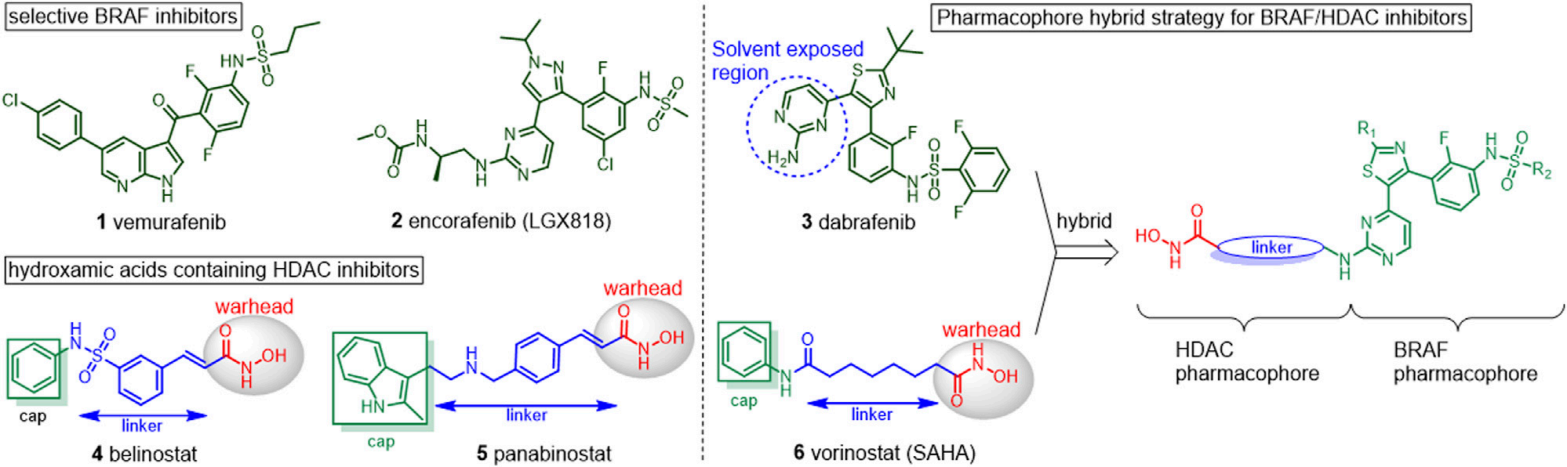
Chemical structures of BRAF inhibitors and HDAC inhibitors and the pharmacophore hybrid strategy for BRAF/HDAC inhibitors.

RAF kinases are key components of the mitogen-activated protein kinase (MAPK) signaling cascade (Wellbrock et al., 2004). Three isoforms of the RAF kinases (i.e., ARAF, BRAF, and CRAF) have been identified, among which BRAF is the most-defined proto-oncogene. BRAF mutations are reported in 6%–8% of all kinds of human cancers, including 50–80% of melanoma, ∼100% of hairy cell leukemia, 45% of papillary thyroid carcinoma, and 11% of colorectal cancer (Davies et al., 2002; Fransén et al., 2004; Ahmadzadeh et al., 2014). More than 90% of the mutations occur on a valine to glutamine at position 600 (BRAF^V600E^), leading to constitutively active BRAF and sustained MAPK activation independent of upstream signaling activity (Chang et al., 2003). Inhibition of mutated BRAF kinase is a well-validated approach for cancer therapy and three selective small molecular BRAF inhibitors, that is, vemurafenib (**1,** Figure 1), encorafenib (**2**), and dabrafenib (**3**) have achieved significant clinical benefits in patients of melanoma and colon cancer harboring BRAF mutations (Bollag et al., 2010; Rheault et al., 2013; Holderfield et al., 2014; Karoulia et al., 2017; Koelblinger et al., 2018). However, almost all patients displayed intrinsical resistance or secondary resistance to these drugs although they also harbor BRAF^V600E^ mutation (Mauri et al., 2021). Dual inhibitors simultaneously targeting BRAF and other critical proliferative pathways were reported to overcome drug resistance or improve efficacy (Kim, 2016; Ma et al., 2018; Palušová et al., 2020; Pinzi et al., 2021).

Histone deacetylases (HDACs) are critical enzymes that regulate the lysine acetylation balance of histones and other proteins (Grunstein, 1997). Dysregulation of HDACs involved in cancer initiation and HDAC inhibition has been proven as an effective therapeutic approach for human malignancies (Marks and Breslow, 2007; Yao and Seto, 2011). To date, five small molecule HDAC inhibitors have been approved for clinical management of hematologic cancers, including the hydroxamic acid–containing belinostat (**4**), panobinostat (**5**), and vorinostat (**6**), macrocyclic romidepsin and benzamide-containing chidamide, while many others are in clinical development (Luan et al., 2019; Ho et al., 2020). However, the clinical indication of HDAC inhibitors is limited in hematologic malignancies, including cutaneous T-cell lymphoma, peripheral T-cell lymphoma (PTCL), and multiple myeloma. HDAC inhibitors alone were not as effective in solid tumors, which severely limits their clinical application (Chen et al., 2020). To overcome these flaws, multitarget or hybrid HDAC inhibitors with better efficacy or ability to overcome drug resistance were thus developed.

Increasing evidence has been reported for the synergistic and additive effects of the joint use of HDAC inhibitors and BRAF inhibitors in colon cancer and melanoma (Lai et al., 2013; Carson et al., 2015; Fu et al., 2019). The combination of PLX4032 and HDAC inhibitors have been displayed complete elimination of cancer cells, and to reduce the progress of resistance to BRAF inhibitors (Madorsky Rowdo et al., 2020). Recently, Emmons et al. revealed that HDAC8 inhibitors could enhance the durability of BRAF inhibitor therapy (Mer et al., 2019). Hence, development of dual inhibitors of BRAF and HDACs may be a valuable strategy to overcome BARFi resistance.

Until now, only one class of phenoxybenzamide compounds has been reported to be BRAF/HDAC1 inhibitors. The IC_50_ values of the optimized compound were 0.073 μM against BRAF V600E and 1.17 μM against HDAC1, respectively (Geng et al., 2019). Previously, we have developed N-(3-ethynyl-2, 4-difluorophenyl) sulfonamide derivatives as selective BRAF inhibitors with potent activities against BRAF-mutant CRC cells, and also reported BRAF/epidermal growth factor receptor (EGFR) dual inhibitors for the treatment of drug-resistant CRCs (Cheng et al., 2014; Li et al., 2015; Li et al., 2016). In a continuing effort to identify BRAFi-based therapeutics for CRC, herein, we designed and synthesized a novel series of hydroxamic acid–containing compounds such as BRAF and HDAC dual-targeted inhibitors. The enzymatic inhibitory activities against BRAF^V600E^, HDAC1/6 and the structure-activity relationship (SAR) study were reported herein.

## 2 Results and Discussion

The pharmacophore hybrid strategy was used for the design of BRAF/HDAC inhibitors by analyzing the canonical pharmacophore of the HDAC inhibitors and the key features of dabrafenib binds to BRAF (Figure 1). As illustrated by vorinostat, the hydroxamic acid–containing HDAC inhibitors share three common structural components: a zinc-binding warhead group, a linker that fits the hydrophobic tunnel, and a cap group that sits outside and interacts with the surface residues of HDAC (Zhang et al., 2018). Importantly, a wide variety of cap groups could be accommodated for HDAC inhibition which allows for the replacement of the cap with another target pharmacophore to design dual-targeted inhibitors. On the other hand, the crystal structure of dabrafenib bound to BRAF^V600E^ revealed its aminopyrimidine moiety pointed toward the solvent-exposed entrance of the pocket, which could accommodate a wide variety of modifications (Rheault et al., 2013; Waizenegger et al., 2016). It was postulated that incorporating the HDAC pharmacophore into this amine group will retain BRAF activity while giving rise to potentially HDAC inhibition activity. Thus, hydroxamic acid and aminopyrimidyl-pharmacophores were tethered via appropriate alkyl linkers, resulting in potential hybridized BRAF/HDAC inhibitors. The alkyl-linker, **R** ^**1**^ substitution on thiazole, and **R** ^**2**^ substitutions on sulfonamide were modified to study the SAR.

**SCHEME 1:**
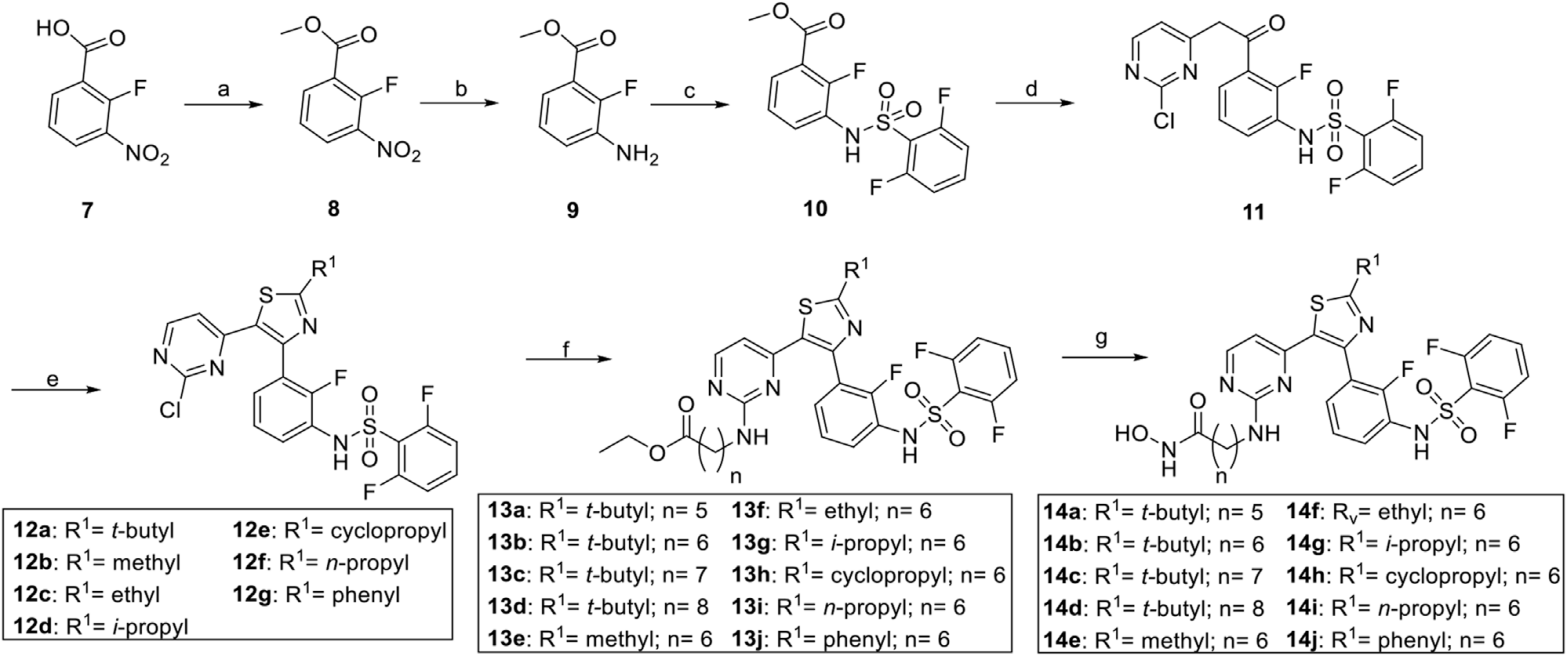
Synthesis of compounds **14a–14j**. ^a^ Reagents and conditions: **(A)** SOCl_2_, MeOH, reflux, 1 h, 87%; **(B)** 10% Pd/C, H_2_, rt, 100%, **(C)** 2,6-difluorobenzenesulfonyl chloride, pyridine, DCM, rt, overnight, 98%; **(D)** 2-chloro-4-methylpyrimidine, LiHMDS, 0°C to rt, 1 h, 92%; **(E)** NBS, 2,2,2-trimethylthioacetamide, DMA, rt to 60°C, 1 h, 30%–44%; **(F)** amines, CsCO_3_, NMP, 60°C, 17 h, 42%–60%; **(G)** NH_2_OH/KOH in MeOH, 0°C, 0.25 h, 50%–74%.

**SCHEME 2:**
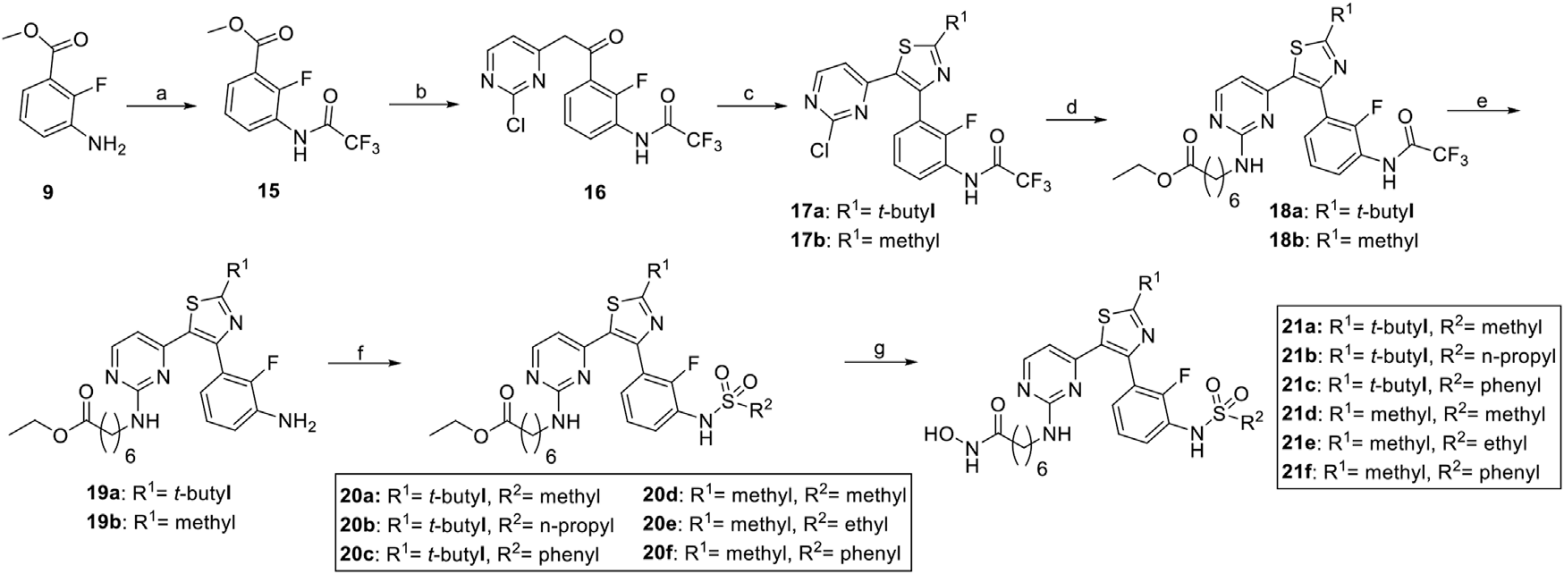
Synthesis of compounds **21d–21f**.^a^ Reagents and conditions: **(A)** rifluoroacetic anhydride, DCM, rt, overnight, 1 h, 88%; **(B)** 2-chloro-4-methylpyrimidine, LiHMDS, 0°C to rt, 1 h, 90%; **(C)** NBS, 2,2,2-trimethylthioacetamide, DMA, rt to 60°C, 1 h, 40%; **(D)** 7-amino-heptanoic acid ethyl ester hydrochloride, CsCO_3_, NMP, 60°C, 17 h, 42%; **(E)** HCl in EtOH, 60°C, 2 h, 94%; **(F)** sulfonyl chlorides, pyridine, DCM, rt, overnight, 74–91%; **(G)** NH_2_OH/KOH in MeOH, 0°C, 0.25 h, 61%–84%.

The synthesis of compounds began with the esterification of 2-fluoro-3-nitrobenzoic acid **(7**) and is detailed in Scheme 1 and Scheme 2. Subsequent nitroreduction of **8** and then replacement of NH_2_ with 2,6-difluorobenzenesulfonyl chloride obtained the sulfonamide compound **10**. Intermediate **10** was then condensed with the lithium anion of 2-chloro-4-methylpyrimidine to generate ketone **11**. Bromination of **11** with N-bromosuccinimide (NBS) followed by cyclization with thioamides afforded the desired thiazole cores **12a–12g**, which were then tethered with different amines to give rise to compounds **13a**–**13j**. The ethyl ester was reacted with hydroxylamine in the presence of potassium hydroxide (KOH) in MeOH to obtain the desired compounds **14a**–**14j** with good yields (Liang et al., 2019). An alternative synthesis route was used for the convenient replacement of the **R** ^**2**^ group. Initially, intermediate **9** was protected by the trifluoroacetyl group. Then, a two-step synthesis of condensation and cyclization afforded the desired thiazole cores **17a** and **17b**. Following the nucleophilic substitution, intermediates **18a** and **18b** were obtained. After deprotection of the trifluoroacetyl under the conditions of HCl in MeOH, the corresponding amines **19a** and **19b** were further reacted with different sulfonyl chlorides to yield **20a–20f**. The hydroxamic acid warhead group was introduced at the last step to afford the targeted compounds **21a–21f**.

The BRAF^V600E^, HDAC1, and HDAC6 enzymatic inhibitory activities of novel compounds are shown in Table 1. Vorinostat and dabrafenib were used as references. Initially, we synthesized compounds with dabrafenib as cap and different *n*-alkyl linkers ranging from five carbons (C_5_, **14a**) to eight carbons (C_8_, **14d**). Unsurprisingly, all these analogs exhibited potent BRAF inhibition with IC_50_ values ranging from 1.71 nM to 3.82 nM. On the other hand, different linker groups resulted in clear SAR in HDAC1/6 activities. A shorter linker, as in **14a** (C_5_), brought the cap group and hydroxamate acid too close together and led to significantly reduced potency against HDAC1 and HDAC6. Compound **14b** with a C_6_ linker had the most potent HDAC6 inhibition with an IC_50_ of 49.20 nM. Further linker extension to C_7_ (**14c**) or C_8_ (**14d**) led to a decrease in HDAC6 activity (IC_50_ = 127.92 nM and IC_50_ = 321.80 nM, respectively). HDAC1 inhibitory activities of **14a**–**14d** showed a similar trend. However, C_7_ compound **14c** (IC_50_ = 312.14 nM) was slightly more potent than C_6_ compound (**14b**, IC_50_ = 493.95 nM). We then fixed the linker to be C_6_ and sought to perform structural modifications on **R** ^**1**^ and **R** ^**2**^ groups.

**TABLE 1:** Enzymatic and cellular activities of compounds.^a^ ^,^ ^b^

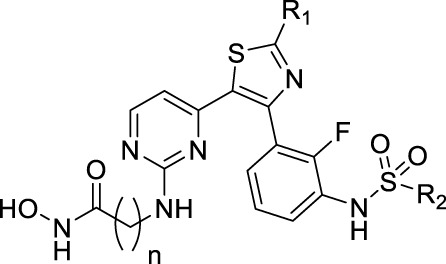								
Cmpd	C_n_	R^1^	R^2^	Enzymatic IC_50_ (nM)			Cellular IC_50_ (μM)	
				BRAF^V600E^	HDAC1	HDAC6	HCT116	HT-29
Vorinostat			NA	50.30	24.09	0.68	3.10	
Dabrafenib			1.11	NA	NA	>10	0.21	
**14a**	5	*t*-Butyl	2,6-Difluorophenyl	3.25	678.50	1211.07	3.00	1.00
**14b**	6	t-Butyl	2,6-Difluorophenyl	1.71	493.95	49.20	1.68	0.31
**14c**	7	*t*-Butyl	2,6-Difluorophenyl	2.27	312.14	127.92	4.47	0.88
**14d**	8	*t*-Butyl	2,6-Difluorophenyl	3.82	765.39	321.80	3.66	0.48
**14e**	6	Methyl	2,6-Difluorophenyl	3.50	42.25	14.60	7.53	6.39
**14f**	6	Ethyl	2,6-Difluorophenyl	1.66	61.30	26.50	4.27	1.27
**14g**	6	*i*-Propyl	2,6-Difluorophenyl	1.51	129.8	30.01	2.68	0.99
**14h**	6	Cyclopropyl	2,6-Difluorophenyl	1.31	49.84	27.10	4.71	2.21
**14i**	6	*n*-Propyl	2,6-Difluorophenyl	1.07	104.50	64.60	3.08	0.97
**14j**	6	Phenyl	2,6-Difluorophenyl	5.14	15.59	140.20	1.08	0.95
**21a**	6	*t*-Butyl	Methyl	1.03	112.55	22.17	8.52	5.02
**21b**	6	*t*-Butyl	*n*-Propyl	1.71	432.07	72.07	5.44	0.78
**21c**	6	*t*-Butyl	Phenyl	1.26	474.40	100.70	2.06	0.11
**21d**	6	Methyl	Methyl	258.00	71.04	21.81	>10	>10
**21e**	6	Methyl	Ethyl	107.49	52.25	18.82	9.15	>10
**21f**	6	Methyl	Phenyl	3.92	49.43	18.68	4.41	4.96

a Kinase activity assays were performed by a FRET-based Z′-Lyte assay.

b Antiproliferative activities were evaluated using a CCK-8, assay. Data are means of three independent experiments.

Aliphatic (methyl (**14e**), ethyl (**14f**), *i*-propyl (**14g**), cyclopropyl (**14h**), *n*-propyl (**14i**)), or phenyl (**14j**) substitutions on the 2-thiozole (**R** ^**1**^) had no dramatic influence on BRAF^V600E^ inhibition. With reference to their HDAC inhibitory activities, these compounds were generally more potent than **14b** against HDAC1. The SAR indicated aromatic and aliphatic **R** ^**1**^ with small steric hindrance was beneficial. The sulfonamide substitution (**R** ^**2**^) was shown to be very important for BRAF inhibition (Li et al., 2015). With compounds **21a**–**21f**, when the **R** ^**1**^ group was *t*-butyl, aliphatic or phenyl substitution of sulfonamide exhibited comparable potency to dabrafenib and **14b**. On the other hand, methyl, the methyl (**21d**) or ethyl (**21e**) sulfonamide group led to a significant decrease in BRAF inhibition. Compounds with the **R** ^**1**^ group of methyl (**14h** and **21d**–**21f**) displayed overall increased activities against HDACs1/6 than compounds with the **R** ^**1**^ group of *t*-butyl (**14b** and **21a**–**21c**). Next, we evaluated the efficacy of dabrafenib, **14b**, **14j**, and **21c** for their inhibition of wild-type BRAF (Table 2). It was found dabrafenib, **14b**, **14j**, and **21c** exerted nanomolar range IC_50_ against wild-type BRAF, with IC_50_ of 2.44 nM, 4.07 nM, 3.86 nM, and 2.86 nM, respectively. Dabrafenib, **14b**, and **21c** displayed week (about 2-fold) selectivity of BRAF^V600E^ over wild-type BRAF, while **14j** with **R** ^**1**^ of phenyl was slightly more potent against wild-type BARF.

**TABLE 2:** Enzymatic activity of dabrafenib, **14b**, **14j** and **21c** against wild-type BRAF kinase.

Compound	BRAF^wt^ enzymatic IC_50_ (nM)
Dabrafenib	2.44
**14b**	4.07
**14j**	3.86
**21c**	2.86

Given the potent BRAF^V600E^ and HDAC1/6 inhibitory activities, the antiproliferation effects of novel inhibitors were investigated using two human colorectal adenocarcinoma cell lines (HT-29 cells harboring BRAF^V600E^ and HCT116 cells with wild-type BRAF) by a Cell Counting Kit-8 (CCK-8) assay (Li et al., 2015). The results revealed that dabrafenib showed better antiproliferative activity against HT-29 cells than that of HCT116 cells. HDAC inhibitor vorinostat inhibited the proliferation of both HCT116 and HT-29 cells, with IC_50_ of 0.68 μM and 3.1 μM, respectively. Most of the novel dual inhibitors exhibited cell growth inhibitory activity except for compounds **21d** and **21e**. The overall results indicated that the BRAF/HDAC inhibitors were broadly efficacious compared to the selective BRAF inhibitor alone. Although **14f**–**14h** and **21a** displayed improved BRAF and HDAC1/6 activities and some other compounds displayed better efficacy against HDAC1/6 enzyme, this did not lead to improved cellular activities compared to **14b** or **21c**. Among the novel inhibitors, **14b** displayed good antiproliferative activities with IC_50_ of 1.68 μM against HCT116 cells and 0.31 μM against HT-29 cells, respectively. Noticeably, it is well-documented that the electron-withdrawing fluorine substitution of phenyl ring reduced the liability to oxidative metabolism (Puszkiel et al., 2019). Compared to **21c** (**R** ^**2**^ = phenyl) which also exerts good cellular activity, **14b** with the **R** ^**2**^ of 2,6-difluorophenyl is superior in the metabolic setting. Therefore, **14b** was selected for further studies.

To elucidate the efficacy and selectivity against other enzymes of HDAC family, compound **14b** was tested against 8 HDAC isoforms (Table 3) together with vorinostat as a positive control (de Ruijter et al., 2003). Compound **14b** showed IC_50_ against class I isoforms HDAC1, HDAC2, HDAC3, and HDAC8 of 0.493 μM, 0.747 μM, 0.913 μM, and 3.004 μM, respectively. HDAC8 is a promising therapeutic anticancer target that modifies non-histone proteins such as p53 (Spreafico et al., 2020). Compounds **14j** and **21c** exerted IC_50_ of 0.876 μM and 0.623 μM against HDAC8 respectively, which was 3.4- and 4.8-fold more potent than **14b** (Supplementary Table S1), indicating that the subtle structural difference may change the selectivity between isomers of HDAC. The inhibition of **14b** against HDAC6 (class IIB) was > 10-fold more potent than class I isoforms and > 800-fold more potent than class IIA HDACs (HDAC4, HDAC5, and HDAC7). Overall, **14b** was a pan-HDAC inhibitor similar to vorinostat, displaying weak selectivity for HDAC6. HDAC6 is the key regulator in oncogenic cell transformation by targeting several non-histone cytoplasmic proteins including Hsp90, α-tubulin, cortactin, HSF1 (Li et al., 2018). The inhibition of class I HDACs and HDAC6 were reported to decrease cell motility and induce apoptosis in cancerous cells, partially contributing to the broadly antitumor effect of **14b** (Miyake et al., 2016; Pulya et al., 2021).

**TABLE 3:** Selectivity of **14b** and vorinostat in HDAC enzyme.

	Enzyme inhibition (μM)							
Compound	Class I				Class IIA			Class IIB
	HDAC1	HDAC2	HDAC3	HDAC8	HDAC4	HDAC5	HDAC7	HDAC6
**14b**	0.493	0.747	0.913	3.004	>100	52.850	43.290	0.049
Vorinostat	0.030	0.091	0.056	1.380	>100	>100	89.200	0.014

**FIGURE 2:**
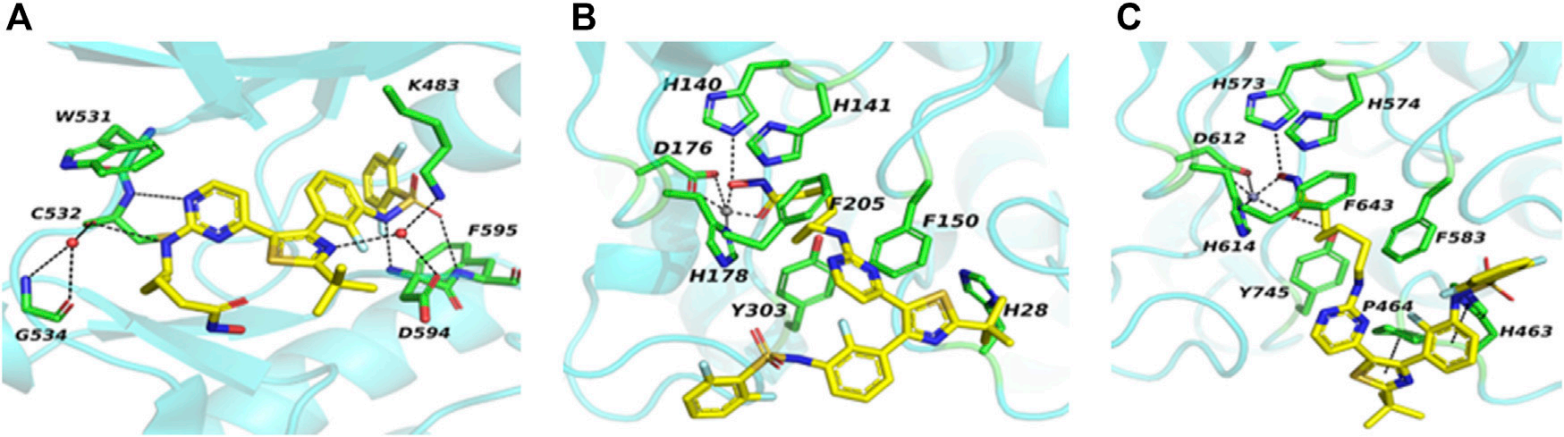
Predicted binding mode of **14b** into BRAF^V600E^ [**(A)**, PDB code: 4xv2], HDAC1 [**(B)**, PDB code: 4bkx] and HDAC6 [**(C)**, PDB code: 5eei]. The targeted proteins are shown in the turquoise picture with selected residues in stick and carbon atoms in green. Water molecules and metal zinc are shown as red and gray spheres, respectively.

In an attempt to understand the interaction between **14b** and its targets, it was docked into the BRAF^V600E^, HDAC1, and HDAC6 (Butler et al., 2010; Millard et al., 2013; Rheault et al., 2013). As shown in Figure 2A, the hydroxamic acid and linker were pointed to the entrance of the ATP pocket of BRAF^V600E^. The aminopyrimidine core of **14b** formed two hydrogen bonds with the backbone amide of C532. Two more hydrogen bonds were formed between the ligand and the residues D594 and F595. In addition, two water-bridged hydrogen-bonding networks were found between the residues C532, G534; the residues K483, D594; and the ligand, respectively. The multiple hydrogen bonds indicated a crucial and strong binding between the inhibitor and BRAF^V600E^. On the docking mode of **14b** to HDAC1 (Figure 2B), the dabrafenib moiety of **14b** projected outside toward the solvent, loosely contacting the hydrophobic residues on the protein surface. The C6 linker was passed through the long tubular channel and stacked between F205 and F150. Hydroxamic acid moiety entered the active site by chelating the essential catalytic zinc ion and formed a hydrogen bond with H140. The binding of **14b** to HDAC6 showed a similar mode to that of HDAC1 except that one more hydrogen bond between the ligand and Y745, and π–π interactions between the thiazole and aromatic rings of the ligand and the residues P464 and H463, respectively, were formed in HDAC6 (Figure 2C). (Miyake et al., 2016) As a result, though with the metal coordination bond present, the absence of the extra hydrogen bond and the π–π interactions between the ligand and HDAC1 pointed to a critical cause of its reduced activity. Given the satisfied inhibition enzymatically and cellularly, it was selected for further biological studies.

**FIGURE 3:**
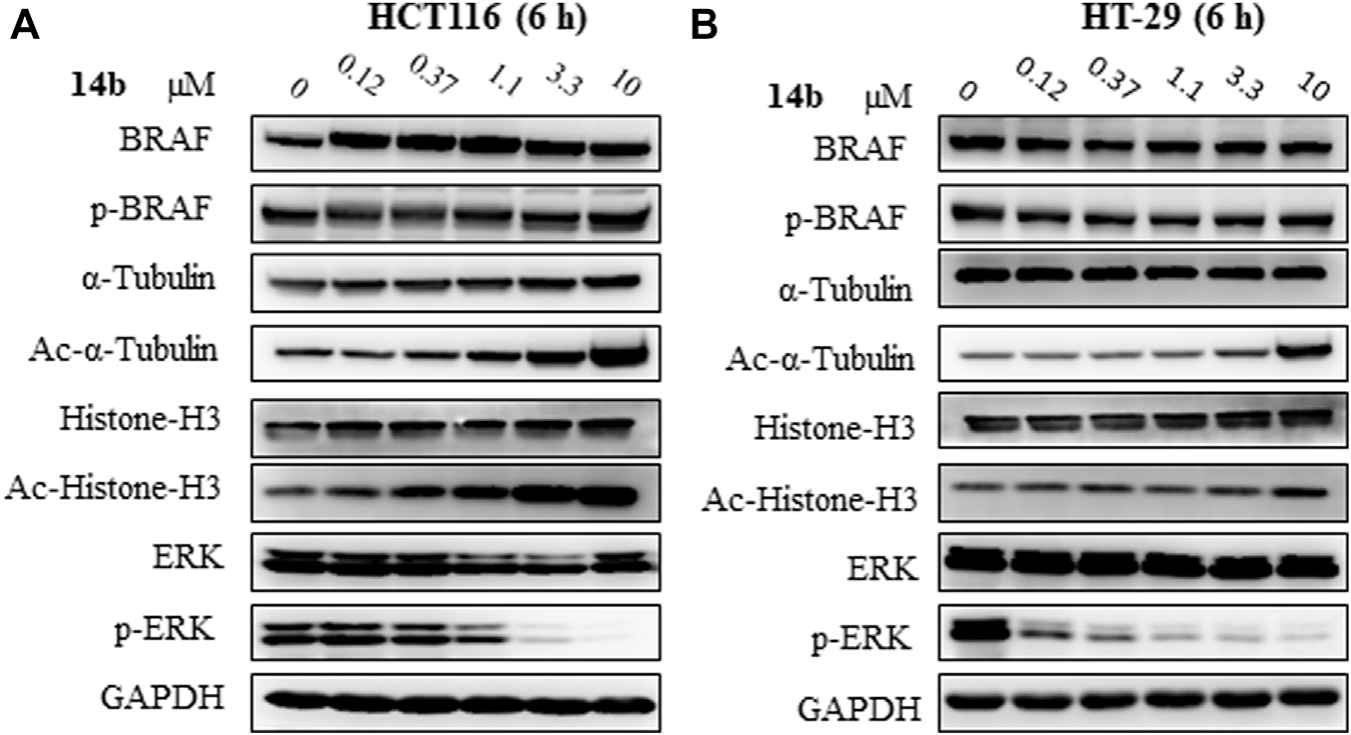
Characterization of dual inhibition of HDAC and RAF pathway. Western blot analysis of **14b** in HCT116 cells **(A)** and in HT-29 cells **(B)**. Cells were treated with 0 μM, 0.12 μM, 0.37 μM, 1.1 μM, 3.3 μM, and 10 μM of drugs and incubated for 6 h. The cell lysis was subjected to Western blot analysis.

Having established compound **14b** as the desirable BRAF/HDAC inhibitor in both BRAF inhibitor sensitive cells and resistant cells, we then proceeded to determine whether or not the compound inhibit dual pathways simultaneously using Western blot analysis. In HT-29 and HCT116 cells, acetylated histone 3 (Ac-histone H3), acetylated α-tubulin (Ac-α-tubulin), and phosphorylated extracellular signal–regulated kinase (p-ERK) were analyzed as effectors of HDAC1, HDAC6, and BRAF signal, respectively (Haggarty et al., 2003). In both cells, the acetylation of α-tubulin and histone H3 was upregulated upon **14b** treatment, indicating the effective inhibition of HDAC6 and HDAC1 signal by **14b** (Figure 3). Phosphorylation of ERK, the downstream effector of BRAF, was significantly inhibited at the lowest concentration of 0.12 μM in HT-29 cells and almost completed inhibition of p-ERK was observed at higher concentrations. It was reported that BRAF inhibitors, including vemurafenib and dabrafenib, induced paradoxical MAPK pathway activation and are contraindicated for the treatment of cancers with RAS mutation (Poulikakos et al., 2010; Adelmann et al., 2016). HCT116 express the mutated active K-RAS (G13D) protein and we found that dabrafenib causes paradoxical activation of ERK in these cells (Supplementary Figure S1). The paradoxical hyperactivation of ERK signaling by BRAFi in BRAF wild-type cells is associated with the emergence of squamous cell carcinoma, a common side-effect of BRAFi (Li et al., 2015; Peng et al., 2015). We were delighted to find that **14b** significantly inhibited phosphorylation of ERK in HCT116 cells, with an effective concentration close to the IC_50_ of HCT116 proliferation (Figure 3A). Similarly, p-ERK inhibition was observed in the presence of **14b** but neither dabrafenib in mouse melanoma B16 cells harbored wild-type BRAF (Supplementary Figure S2). In addition, **14b** did not affect the expression of BRAF itself in both cells. By comparison, vorinostat only upregulated acetylated α-tubulin and acetylated histone H3 in HT-29 cells and HCT116 cells, but had little impact on the p-ERK. On the other hand, dabrafenib reduced the phosphorylation of ERK, but had little impact on the protein acetylation status in HT-29 cells. These results supported that **14b** not only successfully achieved dual inhibition of BRAF and HDAC pathways, but also broke the paradoxical ERK activation in cells with wild-type BRAF.

## 3 Conclusion

An epigenetic malfunction is a common event in most cancer, and HDAC inhibitors were shown to synergize with BRAF/MEK/ERK inhibition (Fu et al., 2019; Maertens et al., 2019). Selective BRAF inhibitors are associated with rapid adaptation and drug resistance as well as cause a high occurrence of secondary skin carcinoma. Developing dual inhibitors of BRAF and HDACs is a valuable strategy to both overcome resistance and enhance effects for BARFi. In summary, we designed a series of hydroxamate acid and 2-aminopyridinyl-containing BRAF/HDAC inhibitors with anti-CRC activity using the pharmacophore hybrid strategy. The compounds potently inhibited BRAF^V600E^, HDAC1, and HDAC6 enzymes and suppressed the proliferation of colorectal cancer cells harboring both wide-type BRAF and V600E mutated BRAF. Furthermore, the representative compound **14b** potently inhibited the activation of the MAPK pathway and upregulated Ac-histone-H3 and Ac-a-tubulin in both cells. Selective BRAF inhibitors vemurafenib and dabrafenib were reported to induce paradoxically upregulation of ERK in HCT116 cells harboring wild-type BRAF. More importantly, the effectiveness of ERK inhibition and cytotoxicity of **14b** in HCT116 cells, suggests its potential as a “paradox breaker” to reduce the side effect related to selective BRAFi. Collectively, our studies provided valuable tool compounds for dual pathway inhibition with a single molecule. BRAF mutations are linked to more advanced and aggressive colorectal cancer, lung cancer, and thyroid carcinoma. So it is worthwhile to explore the efficacy of BRAF/HDAC inhibitors against these types of tumors harboring mutated BRAF. In addition, further studies to elucidate the crosstalk of BRAF/HDAC pathways using tool compounds will be highly worthwhile to explore the clinical potential of the dual inhibitors.

## 4 Materials and Methods

### 4.1 Chemistry

#### 4.1.1 General Conditions

All reagents and solvents were used directly as purchased from commercial sources. Flash chromatography was performed using silica gel (200–300 mesh). All reactions were monitored by thin-layer chromatography (TLC), using silica gel plates with fluorescence F_254_ and UV light visualization. ^1^H NMR spectra were recorded on a Bruker AV-400 spectrometer at 400 MHz. Coupling constants (*J*) are expressed in hertz (Hz). Chemical shifts (δ) of NMR are reported in parts per million (ppm) units relative to an internal control (TMS). Low-resolution ESI–MS was recorded on an Agilent 1200 HPLC-MSD mass spectrometer and high-resolution ESI–MS on an Applied Biosystems Q-STAR Elite ESI–LC–MS/MS mass spectrometer. HPLC instrument, Dionex Summit HPLC (column: Diamonsil C18, 5.0 μM, 4.6 × 250 mm (Dikma Technologies); detector, PDA-100 photodiode array; injector, ASI-100 autoinjector; pump, p-680A). A flow rate of 0.5 ml/min was used with a mobile phase of ACN in H2O with a 0.1% modifier (TFA, v/v).

#### 4.1.2 Synthetic Procedures for 14a–14j

##### 4.1.2.1 Methyl 2-Fluoro-3-Nitrobenzoate (**8**)

Into a 500 ml two-neck round bottom flask was placed 50 g (270 mmol) of 2-fluoro-3-nitrobenzoic acid (**7**) and 270 ml of anhydrous methanol (MeOH). A measure of 29 ml thionyl chloride was added dropwise in an ice bath. Then, the reaction was transferred to an oil bath and heated to reflux for 1 h (hour). After cooling to room temperature (rt), 1-fold of ice-cold water was added to the flask and the mixture was filtered through a Buchner funnel. The filter residue was washed three times with water and dried under reduced pressure to give 47 g (yield: 87%) of the yellowish compound **8**. ^1^H NMR (400 MHz, DMSO-d_6_): *δ* (ppm) 8.28–8.10 (m, 2H), 7.30–7.37 (m, ^1^H), and 3.99 (s, 3H). MS (ESI), m/z: 200 (M^+^ + H^+^).

##### 4.1.2.2 Methyl 3-Amino-2-Fluorobenzoate (**9**)

In a 500 ml round bottom flask was placed 20 g methyl 2-fluoro-3-nitrobenzoate (8) and 200 ml MeOH. The air in the flask was exchanged for argon and then 1 g wet palladium on carbon (Pd/C) was added to the flask. Then, the reaction vessel was filled with hydrogen gas using a balloon and the reaction was stirred at rt. After 6 h, the completion of the reaction was monitored using TLC. The mixture was filtered through celite and washed with extra EtOAc. The filtrate was concentrated under reduced pressure and dried to get 17 g compound **9** as an orange oil (yield: 100%). ^1^H NMR (400 MHz, DMSO-d_6_): *δ* (ppm) 6.92–7.01 (m, 3 H), 5.37 (s, 2 H), and 3.81 (s, 3 H). MS (ESI), m/z: 170 (M^+^ + H^+^).

##### 4.1.2.3 Methyl 3-[(2,6-Difluorophenyl)Sulfonamido]-2-Fluorobenzoate (**10**)

In o a 500 ml flask was placed methyl 3-amino-2-fluorobenzoate (6.5 g, 35.8 mmol) and DCM (150 ml), and pyridine (3.5 ml, 35.8 mmol). Then, 2,6-difluorobenzenesulfonyl chloride (9 g, 42.3 mmol) in DCM (70 ml) was added dropwise via a funnel and the reaction mixture was allowed to stir at room temperature overnight. The reaction mixture was filtered through celite and washed twice with EtOAc. The filtrate was concentrated and purified by column chromatography [PE (petroleum ether): EtOAc = 5:1] to give 12.2 g (98%) of compound **10**. ^1^H NMR (400 MHz, DMSO-d_6_): *δ* (ppm) 10.98 (s, 1 H), 7.64–7.82 (m, 3H), 7.46–7.61 (m, 1 H), 7.29 (t, *J =* 8.8 Hz, 2 H), and 3.81 (s, 3 H). MS (ESI), m/z: 346 (M^+^ + H^+^).

##### 4.1.2.4 N-{3-[2-(2-Chloropyrimidin-4-yl)Acetyl]-2-Fluorophenyl}-2,6-Difluorobenzene-Sulfonamide (**11**)

In a 250 ml flask was placed methyl 3-[(2,6-difluorophenyl)sulfonamido]-2-fluorobenzoate (**10**, 12.2 g, 35.3 mmol) and THF (70 ml). The flask was placed in an ice–water bath and 80 ml of 1M lithium bis(trimethylsilyl)amide (LiHMDS) was added dropwise via an addition funnel and then 2-chloro-4-methylpyrimidine (4.5 g, 35.0 mmol) was added dropwise via syringe. Then, the reaction was allowed to warm to rt and stirred for 4 h. The completion of the reaction was monitored by TLC. The solvent volume was reduced to half under reduced pressure and then treated with 6 N HCl to neutralize the mixture. EtOAc was added and the organic layers were separated. The aqueous layer was extracted twice with EtOAc and the combined organic layer was washed once with brine, dried over Na_2_SO_4_, and concentrated onto silica gel. The residue was purified by column chromatography (PE: EtOAc = 3:1) to give 14 g (92%) of compound **11**. ^1^H NMR (400 MHz, DMSO-d_6_): *δ* (ppm) 13.40 (s, 1 H), 10.82–11.12 (m, 2 H), 8.48–8.87 (m, 2 H), 7.67–7.80 (m, 3 H), 7.61–7.69 (m, 1 H), 7.52–7.61 (m, 2 H), 7.50 (d, *J* = 5.31 Hz, 1 H), 7.43 (td, *J* = 7.60, 1.28 Hz, 1 H), 7.21–7.38 (m, 6 H), 6.13 (s, 1 H), and 4.49 (s, 2 H). MS (ESI), m/z: 442 (M^+^ + H^+^).

##### 4.1.2.5 N-{3-[2-(Tert-Butyl)-5-(2-Chloropyrimidin-4-yl)Thiazol-4-yl]-2-Fluorophenyl}-2,6-Difluorobenzenesulfonamide (**12a**)

In a solution of N-{3-[2-(2-chloropyrimidin-4-yl)acetyl]-2-fluorophenyl}-2,6-difluorobenzenesulfonamide (**11**, 14 g, 32 mmol) in 50 ml N,N-dimethylacetamide (DMA), 6.42 g of N-bromosuccinimide (NBS, 32 mmol) was added via small potions and the solution was allowed to stir for 2 h at rt. Then, 2,2-dimethylpropanethioamide (4.2 g, 32 mmol) was then added. The reaction was heated to 60°C for 2 h. After completion of the reaction, the mixture was diluted with water and extracted two times with EtOAc. The combined EtOAc layers were washed three times with water to remove DMA, dried over Na_2_SO_4_, filtered, and concentrated onto silica gel. The residue was purified by column chromatography (PE: EtOAc = 3:1) to give 7.35 g (yield: 34%) of compound **12a**. ^1^H NMR (400 MHz, DMSO-d6) δ ppm 8.30 (d, *J =* 5.2Hz, ^1^H), 7.72 (t, *J =* 8.0Hz, ^1^H), 7.48–7.55 (m, ^1^H), 7.31–7.36 (m, 2H), 7.22–7.26 (m, ^1^H), 6.99 (t, *J =* 8.8Hz, ^1^H), 6.74 (d,*J =* 5.2Hz, ^1^H), and 1.48 (s, 9H). MS (ESI): 539.1 (M^+^ + H^+^).

##### 4.1.2.6 Ethyl 7-{[4-(2-(Tert-Butyl)-4-(3-((2,6-Difluorophenyl)Sulfonamido)-2-Fluorophenyl)Thiazol-5-yl)Pyrimidin-2-yl]Amino}Heptanoate (**13b**)

In o a 250 ml flask was placed 1.11g of N-{3-[2-(tert-butyl)-5-(2-chloropyrimidin-4-yl)thiazol-4-yl]-2-fluorophenyl}-2,6-difluorobenzenesulfonamide (**12a**, 2 mmol), 1.3g of CsCO_3_ (4 mmol), 0.63 mg of 7-amino-heptanoic acid ethyl ester hydrochloride (3 mmol), and 5 ml of N-methylpyrrolidone (NMP). The reaction mixture was heated to 60°C for 17 h. After cooling to rt, water was added and the mixture was neutralized with dilute hydrochloric acid. The mixture was extracted three times with EtOAc. The combined EtOAc washings were washed two times with water, dried over Na_2_SO_4_, filtered, and concentrated onto silica gel. The residue was purified by column chromatography (PE: EtOAc = 2:1) to give 0.56 g (41.5%) of compound **13b**. ^1^H NMR (400 MHz, DMSO-d_6_): δ ppm 7.90 (s, ^1^H), 7.71 (t, *J =* 8.0Hz, ^1^H), 7.50 (td, *J =* 7.2, 2.4Hz, ^1^H), 7.32 (t, *J =* 6.4Hz, ^1^H), 7.20 (t, *J =* 8.0Hz, ^1^H), 6.98 (t, *J =* 8.4Hz, ^1^H), 6.08 (s, ^1^H), 4.13 (q, *J =* 7.2 Hz, 2H), 3.17–3.27 (m, 2H), 2.30 (t, *J =* 7.2Hz, 2H), 1.55–1.66 (m, 4H), 1.47 (s, 9H), 1.35–1.40 (m, 4H), and 1.25 (t, *J =* 7.2Hz, 3H). MS (ESI), m/z: 675 (M^+^ + H^+^).

##### 4.1.2.7 7-{[4-(2-(Tert-Butyl)-4-(3-((2,6-Difluorophenyl)Sulfonamido)-2-Fluorophenyl)Thiazol-5-yl)Pyrimidin-2-yl]Amino}-N-Hydroxyheptanamide (**14b**)

Compound **14b** was synthesized using the following procedures as previously described.[38] a: preparation of NH_2_OH/KOH in MeOH solution: 9.34 g of hydroxylamine hydrochloride in 48 ml of MeOH was added dropwise to a solution of 11.2 g potassium hydroxide (KOH) in 28 ml MeOH in an ice–water bath. After that, the mixture was stirred for 0.5 h and then filtered. The filtrate was sealed and stored at −20°C for further usage. b: in a 50 ml flask was placed 0.675 g of ethyl 7-{[4-(2-(tert-butyl)-4-(3-((2,6-difluorophenyl)sulfonamido)-2-fluorophenyl)thiazol-5-yl)pyrimidin-2-yl]amino}heptanoate (**13b**, 1 mmol) and 5 ml of MeOH. A measure of 5 ml of NH_2_OH/KOH MeOH solution was added and stirred for 15 min in an ice–water bath. The completion of the reaction was monitored by TLC. Then, 20 ml of water was added and the pH was adjusted to 6 using 1N HCl. The precipitation was filtered and washed three times with water and dried under reduced pressure to obtain compound 500 mg **14b** as light-yellow solid (yield: 74%). ^1^H NMR (500 MHz, DMSO-d_6_): *δ* (ppm) 10.86 (s, ^1^H), 10.32 (s, ^1^H), 8.64 (s, ^1^H), 8.02 (d, *J =* 5.0Hz, ^1^H), 7.65–7.67 (m, ^1^H), 7.42 (t, *J =* 7.0Hz, ^1^H), 7.30–7.35 (m, ^1^H), 7.20–7.28 (m, 4H), 5.80–5.95 (m, ^1^H), 3.05–3.20 (m, 2H), 1.94 (t, *J =* 7.0Hz, ^1^H), 1.45–1.52 (m, 4H), 1.40 (s, 9H), and 1.22–1.37 (m, 4H). HRMS (ESI) calcd for C_30_H_33_F_3_N_6_O_4_S_2_ (M-H)^−^: 661.1884; found 661.1879. HPLC purity = 95%, Rt 27.6 min. Melting point: 95.3°C.

##### 4.1.2.8 6-{[4-(2-(Tert-butyl)-5-(3-(2,6-Difluorophenylsulfonamido)-2-Fluorophenyl)Thiazol-4-yl)Pyrimidin-2-yl]Amino}-N-Hydroxyhexanamide (**14a**)

Yield: 50%. ^1^H NMR (400 MHz, DMSO-d_6_): *δ* (ppm) 10.84 (s, ^1^H), 10.31 (s, ^1^H), 8.02 (s, ^1^H), 7.64–7.71 (m, ^1^H), 7.42 (t, *J =* 7.0Hz, ^1^H), 7.33–7.35 (m, ^1^H), 7.20–7.30 (m, 4H), 5.78–6.00 (brs, ^1^H), 3.10–3.20 (m, 2H), 1.95 (t, *J =* 7.2Hz, 2H), 1.49–1.53 (m, 4H), and 1.24–1.28 (m, 2H). HRMS (ESI) calcd for C_29_H_31_F_3_N_6_O_4_S_2_ (M-H)^−^: 647.1727; found 647.1774.

##### 4.1.2.9 8-{[4-(2-(Tert-Butyl)-4-(3-(2,6-Difluorophenylsulfonamido)-2-Fluorophenyl)Thiazol-5-yl)Pyrimidin-2-yl]Amino}-N-Hydroxyoctanamide (**14c**)

Yield: 62%. ^1^H NMR (400 MHz, DMSO-d_6_): *δ* (ppm) 10.86 (s, ^1^H), 10.31 (s, ^1^H), 8.64 (s, ^1^H), 8.02 (d, *J =* 4.0Hz, ^1^H), 7.66–7.68 (m, ^1^H), 7.41–7.44 (m, ^1^H), 7.34–7.40 (m, ^1^H), 7.21–7.28 (m, 4H), 5.76–5.91 (brs, ^1^H), 3.13–3.17 (m, 2H), 1.93 (t, *J =* 6.0Hz, 2H), 1.47–1.53 (m, 4H), 1.40 (s, 9H), and 1.20–1.27 (m, 6H). HRMS (ESI) calcd for C_3_^1^H_35_F_3_N_6_O_4_S_2_ (M-H)^+^: 677.2186; found 677.2177.

##### 4.1.2.10 9-{[4-(2-(Tert-Butyl)-4-(3-(2,6-Difluorophenylsulfonamido)-2-Fluorophenyl)Thiazol-5-yl)Pyrimidin-2-yl]Amino}-N-Hydroxynonanamide (**14d**)

Yield: 71%. ^1^H NMR (400 MHz, DMSO-d_6_): *δ* (ppm) 10.86 (s, ^1^H), 10.31 (s, ^1^H), 8.6 3 (s, ^1^H), 8.02 (d, *J =* 4Hz, ^1^H), 7.60–7.69 (m, ^1^H), 7.42 (t, *J =* 5.6Hz, ^1^H), 7.20–7.31 (m, 5H), 5.78–5.88 (brs, ^1^H), 3.10–3.20 (m, 2H), 1.92 (t, *J =* 6.0Hz, 2H), 1.47–1.50 (m, 4H), 1.40 (s, 9H), and 1.20–1.30 (m, 8H). HRMS (ESI) calcd for C_32_H_37_F_3_N_6_O_4_S_2_ (M-H)^−^: 689.2197; found 689.2190.

##### 4.1.2.11 7-{[4-(4-(3-(2,6-Difluorophenylsulfonamido)-2-Fluorophenyl)-2-Methylthiazol-5-yl)Pyrimidin-2-yl]Amino}-N-Hydroxyheptanamide (**14e**)

Yield: 71%. ^1^H NMR (400 MHz, DMSO-d_6_): *δ* (ppm) 10.88 (s, ^1^H), 10.32 (s, ^1^H), 8.65 (s, ^1^H), 8.02 (d, *J =* 4.4Hz, ^1^H), 7.63–7.72 (m, ^1^H), 7.38–7.45 (m, ^1^H), 7.20–7.36 (m, 5H), 5.80–5.92 (brs, ^1^H), 3.08–3.20 (m, 2H), 2.68 (s, 3H), 1.94 (t, *J =* 7.0Hz, 2H), 1.43–1.53 (m, 4H), and 1.22–1.30 (m, 4H). HRMS (ESI) calcd for C_27_H_27_F_3_N_6_O_4_S_2_ (M-H)^−^: 619.1414; found 619.1409.

##### 4.1.2.12 7-{[4-(4-(3-(2,6-Difluorophenylsulfonamido)-2-Fluorophenyl)-2-Ethylthiazol-5-yl)Pyrimidin-2-yl]Amino}-N-Hydroxyheptanamide (**14f**)

Yield: 65%. ^1^H NMR (400 MHz, DMSO-d_6_): *δ* (ppm) 10.86 (s, ^1^H), 10.31 (s, ^1^H), 8.02 (d, *J =* 4.8Hz, ^1^H), 7.64–7.71 (m, ^1^H), 7.42 (t, *J* = 7.5Hz, ^1^H), 7.35 (t, *J* = 6.7Hz, ^1^H), 7.21–7.30 (m, 4H), 5.80–5.95 (brs, ^1^H), 3.10–3.20 (m, 2H), 3.02 (q, *J =* 7.6Hz, 2H), 1.94 (t, *J =* 7.2Hz, 2H), 1.43–1.55 (m, 4H), 1.32 (t, *J =* 7.6Hz, 3H), and 1.27 (m, 4H). HRMS (ESI) calcd for C_28_H_29_F_3_N_6_O_4_S_2_ (M-H)^−^:633.1571; found 633.1571.

##### 4.1.2.13 7-{[4-(4-(3-(2,6-Difluorophenylsulfonamido)-2-Fluorophenyl)-2-Isopropylthiazol-5-yl)Pyrimidin-2-yl]Amino}-N-Hydroxyheptanamide (**14g**)

Yield: 70%. ^1^H NMR (400 MHz, DMSO-d_6_): *δ* (ppm) 10.87 (s, ^1^H), 10.32 (s, ^1^H), 8.65 (s, ^1^H), 8.02 (d, *J =* 5.5Hz, ^1^H), 7.65–7.70 (m, ^1^H), 7.43 (t, *J =* 7.5Hz, ^1^H), 7.35 (t, *J =* 6.7Hz, ^1^H), 7.22–7.29 (m, 4H), 5.74–5.94 (m, ^1^H), 3.27–3.30 (m, ^1^H), 3.06–3.20 (m, 2H), 1.94 (t, *J =* 7.5Hz, 2H), 1.47–1.52 (m, 4H), 1.35 (d, *J =* 7.0Hz, 6H), and 1.25–1.30 (m, 4H). HRMS (ESI) calcd for C_29_H_31_F_3_N_6_O_4_S_2_ (M-H)^−^: 647.1727; found 647.1774.

##### 4.1.2.14 7-{[4-(2-Cyclopropyl-4-(3-(2,6-Difluorophenylsulfonamido)-2-Fluorophenyl)Thiazol-5-yl)Pyrimidin-2-yl]Amino}-N-Hydroxyheptanamide (**14h**)

Yield: 54%. ^1^H NMR (400 MHz, DMSO-d_6_): *δ* (ppm) 10.88 (s, ^1^H), 10.33 (s, ^1^H), 8.04 (d, *J =* 4.4Hz, ^1^H), 7.66–7.70 (m, ^1^H), 7.34–7.45 (m, 3H), 7.20–7.30 (m, 4H), 5.80–6.00 (brs, ^1^H), 3.00 (m, 2H), 1.94 (m, 2H), 1.49 (m, 4H), and 1.27–1.34 (m, 9H). HRMS (ESI) calcd for C_29_H_29_F_3_N_6_O_4_S_2_ (M-H)^−^: 645.1571; found 645.1514.

##### 4.1.2.15 7-{[4-(4-(3-(2,6-Difluorophenylsulfonamido)-2-Fluorophenyl)-2-Propylthiazol-5-yl)Pyrimidin-2-yl]Amino}-N-Hydroxyheptanamide (**14i**)

Yield: 67%. ^1^H NMR (400 MHz, DMSO-d_6_): *δ* (ppm) 10.86 (s, ^1^H), 10.32 (s, ^1^H), 8.64 (s, ^1^H), 8.02 (d, *J =* 4.8Hz, ^1^H), 7.63–7.71 (m, ^1^H), 7.43 (t, *J =* 7.2Hz, ^1^H), 7.34 (t, *J =* 6.0Hz, ^1^H), 7.20–7.29 (m, 4H), 5.80–5.60 (m, ^1^H), 3.10–3.25 (m, 2H), 2.95 (t, *J =* 7.6Hz, 2H), 1.94 (t, *J =* 7.2Hz, 2H), 1.70–1.80 (m, 2H), 1.40–1.52 (m, 4H), 1.22–1.30 (m, 4H), and 0.97 (t, *J =* 7.6Hz, 3H). HRMS (ESI) calcd for (M-H)^−^: 647.1728; found 647.1730.

##### 4.1.2.16 7-{[4-(4-(3-(2,6-Difluorophenylsulfonamido)-2-Fluorophenyl)-2-Phenylthiazol-5-yl)Pyrimidin-2-yl]Amino}-N-Hydroxyheptanamide (**14j**)

Yield: 59%. ^1^H NMR (400 MHz, DMSO-d_6_): *δ* (ppm) 10.93 (s, ^1^H), 10.33 (s, ^1^H), 8.65 (s, ^1^H), 8.07 (d, *J =* 5.2Hz, ^1^H), 7.88–8.02 (m, 2H), 7.60–7.70 (m, ^1^H), 7.53–7.58 (m, 3H), 7.47 (t, *J =* 7.2Hz, ^1^H), 7.38–7.42 (m, ^1^H), 7.25–7.33 (m, 2H), 7.23 (t, *J =* 9.0Hz, 2H), 5.85–6.00 (brs, ^1^H), 3.15–3.25 (m, 2H), 1.95 (t, *J =* 7.2Hz, 2H), 1.47–1.57 (m, 4H), and 1.25–1.35 (m, 4H). HRMS (ESI) calcd for C_32_H_29_F_3_N_6_O_4_S_2_ (M-H)^−^: 681.1571; found 681.1558.

#### 4.1.3 Synthetic Procedures for **21a**–**21f**

##### 4.1.3.1 Methyl 2-Fluoro-3-(2,2,2-Trifluoroacetamido)Benzoate (**15**)

In a 250 ml round bottom flask was added 10 g of methyl 3-amino-2-fluorobenzoate (**9**, 60 mmol) in 180 ml dry tetrahydrofuran (HTF). Then, 5.8 ml of trifluoroacetic anhydride (72 mmol), 12.5 ml of triethylamine (Et_3_N, 90 mmol), and 73 mg of 4-dimethylaminipyridine (DMAP) were successively added and the reaction was stirred for 3 h. The mixture was concentrated under reduced pressure. The residue was washed with DCM/PE (1:1) and filtered. The filtrate was further concentrated on silica gel and purified by column chromatography (PE: EtOAc = 7:1). Combined filtered solid and chromatographic elution obtained 11.7 g (90%) of compound **15**. ^1^H NMR (400 MHz, DMSO-d_6_): *δ* (ppm) .8.32–8.17 (m, 1 H), 7.76–7.70 (m, ^1^H), 7.30 (t, *J =* 7.6Hz, ^1^H), and 3.91 (s, 3 H). MS (ESI), m/z: 266 (M^+^ + H^+^).

##### 4.1.3.2 N-{3-[2-(2-Chloropyrimidin-4-yl)Acetyl]-2-Fluorophenyl}-2,2,2-Trifluoroacetamide (**16**)

In a three-neck 250 ml round bottom flask was placed 11.7 g of methyl 2-fluoro-3-(2,2,2-trifluoroacetamido)benzoate (**15**, 53 mmol) and 100 ml of THF (70 ml). The flask was placed in an ice–water bath and 132 ml 1M LiHMDS was added dropwise via an addition funnel and then 6.8 g 2-chloro-4-methylpyrimidine (53 mmol) via syringe. After the addition was complete, the reaction was allowed to warm to rt and stirred for 4 h. The completion of the reaction was monitored by TLC. The solvent volume was reduced to half under reduced pressure and then treated with 6 N HCl to neutralize the mixture. The solution was extracted three times with EtOAc and the combined organic layer was washed once with brine, dried over Na_2_SO_4_, and concentrated onto silica gel. The residue was purified by column chromatography (PE: EtOAc = 3:1) to give 18.5 g (yield: 98%) of compound **16**. ^1^H NMR (400 MHz, DMSO-d_6_): *δ* (ppm) 13.80 (s, ^1^H), 8.46 (d, *J =* 5.2Hz, ^1^H), 8.32 (t, *J =* 7.6Hz, ^1^H), 8.14–8.21 (m, ^1^H), 7.76 (t, *J =* 7.6Hz, ^1^H), 7.32 (t, *J =* 7.6Hz, ^1^H), 6.95 (d, *J =* 5.2Hz, ^1^H), and 6.17 (s, ^1^H). MS (ESI), m/z: 362 (M^+^ + H^+^).

##### 4.1.3.3 N-{3-[2-(Tert-Butyl)-5-(2-Chloropyrimidin-4-yl)Thiazol-4-yl]-2-Fluorophenyl}-2,2,2-Trifluoroacetamide (**17a**)

In a solution of 5 g of N-(3-(2-(2-chloropyrimidin-4-yl)acetyl)-2-fluorophenyl)-2,2,2-trifluoroacetamide (**16**, 14 mmol) in 50 ml of DMA, 2.5 g NBS (14 mmol) was added and the solution and was allowed to stir for 2 h at rt. Then, 1.05 g of 2,2-dimethylpropanethioamide (14 mmol) was added at rt. The reaction was heated to 60°C for 2 h. After completion of the reaction, the mixture was diluted with water and extracted two times with EtOAc. The combined EtOAc washings were washed three times with water to remove DMA, dried over Na_2_SO_4_, filtered, and concentrated onto silica gel. The residue was purified by column chromatography (PE: EtOAc = 3:1) to give 4 g (yield: 68%) of compound **17a**. ^1^H NMR (400 MHz, CDCl_3_): *δ* (ppm) 8.41 (d, *J =* 5.6Hz, ^1^H), 8.36 (td, *J =* 7.6, 1.2Hz, ^1^H), 8.09 (s, ^1^H), 7.43 (td, *J =* 7.2, 1.2Hz, ^1^H), 7.35 (t, *J =* 8.0Hz, ^1^H), 6.92 (d, *J =* 5.5Hz, ^1^H), and 1.50 (s, 9H). MS (ESI), m/z: 457 (M^+^ + H^+^).

##### 4.1.3.4 Ethyl 7-{[4-(2-(Tert-Butyl)-4-(2-Fluoro-3-(2,2,2-Trifluoroacetamido)Phenyl)Thiazol-5-yl)Pyrimidin-2-yl]Amino}Heptanoate (**18a**)

In a 250 ml flask was placed 4 g of N-{3**-[2-**(tert-butyl)-5-(2-chloropyrimidin-4-yl)thiazol-4-yl]-2-fluorophenyl}-2,2,2-trifluoroacetamide (1**7**, 10 mmol), 9.8 g of CsCO_3_ (30 mmol), 3 g of 7-amino-heptanoic acid ethyl ester hydrochloride (15 mmol), and 40 ml of NMP. The reaction mixture was heated to 60°C for 17 h. After cooling to rt, water was added and dilute hydrochloric acid was then added to neutralize the mixture. The mixture was extracted three times with EtOAc. The combined EtOAc washings were washed two times with water, dried over Na_2_SO_4_, filtered, and concentrated onto silica gel. The residue was purified by column chromatography (PE: EtOAc = 3:1) to give 3.6 g (65%) of compound **18a**. ^1^H NMR (400 MHz, DMSO-d_6_): *δ* (ppm) 7.95–8.03 (m, ^1^H), 7.67 (t, *J =* 7.6Hz, ^1^H), 7.36 (t, *J =* 6.8Hz, ^1^H), 7.23 (t, *J =* 8.0Hz, ^1^H), 6.21 (d, *J =* 5.2Hz, ^1^H), 4.12 (q, *J =* 7.2Hz, 2H), 3.23–3.45 (m, 2H), 2.76 (m, ^1^H), 2.29 (t, *J =* 7.6Hz, 2H), 1.53–1.67 (m, 4H), 1.45–1.52 (m, 4H), 1.41 (s, 9H), and 1.24 (t, *J =* 7.2Hz, 2H). MS (ESI), m/z: 596 (M^+^ + H^+^).

##### 4.1.3.5 Ethyl 7-{[4-(4-(3-Amino-2-Fluorophenyl)-2-(Tert-Butyl)Thiazol-5-yl)Pyrimidin-2-yl]Amino}Heptanoate (**19a**)

A measure of 3.6 g ethyl of 7-{[4-(2-(tert-butyl)-4-(2-fluoro-3-(2,2,2-trifluoroacetamido)phenyl)thiazol-5-yl)pyrimidin-2-yl]amino}heptanoate (**18a**) was dissolved in 60 ml HCl/EtOH solution and heated to 60°C for 2 h. The completion of the reaction was monitored by TLC. The solvent was removed under reduced pressure. The product was used in the next step without further purification. ^1^H NMR (400 MHz, DMSO-d_6_): *δ*(ppm) 7.95–8.03 (m, ^1^H), 7.67 (t, *J* = 7.6Hz, ^1^H), 7.36 (t, *J* = 6.8Hz, ^1^H), 7.23 (t, *J* = 8.0Hz, ^1^H), 6.21 (d, *J* = 5.2Hz, ^1^H), 4.12 (q, *J* = 7.2Hz, 2H), 3.23–3.45 (m, 2H), 2.76 (m, ^1^H), 2.29 (t, *J* = 7.6Hz, 2H), 1.53–1.67 (m, 4H), 1.45–1.52 (m, 4H), 1.41 (s, 9H), and 1.24 (t, *J* = 7.2Hz, 2H). MS(ESI), m/z: 458 (M^+^ + H^+^).

##### 4.1.3.6 7-{[4-(2-(Tert-Butyl)-4-(2-Fluoro-3-(Methylsulfonamido)Phenyl)Thiazol-5-yl)Pyrimidin-2-yl]Amino}-N-Hydroxyheptanamide (**21a**)

Yield: 61%. ^1^H NMR (400 MHz, DMSO-d_6_): *δ* (ppm) 10.32 (s, ^1^H), 9.65–9.75 (brs, ^1^H), 8.65 (s, ^1^H), 8.10 (d, *J =* 5.2Hz, ^1^H), 7.51 (t, *J =* 7.5Hz, ^1^H), 7.30–7.36 (m, ^1^H), 7.28–7.31 (m, ^1^H), 7.23–7.26 (m, ^1^H), 6.00–6.20 (m, ^1^H), 3.07–3.20 (m, 2H), 2.98 (s, 3H), 1.93 (t, *J =* 6.0Hz, ^1^H), 1.55–1.63 (m, 4H), 1.43 (s, 9H), and 1.20–1.30 (m, 4H). HRMS (ESI) calcd C_25_H_33_FN_6_O_4_S_2_ for (M-H)^−^:563.1916; found 563.1910.

##### 4.1.3.7 7-{[4-(2-(Tert-Butyl)-4-(2-Fluoro-3-(Propylsulfonamido)Phenyl)Thiazol-5-yl)Pyrimidin-2-yl]Amino}-N-Hydroxyheptanamide (**21b**)

Yield: 70%. ^1^H NMR (400 MHz, DMSO-d_6_): *δ* (ppm) 10.34 (s, ^1^H), 9.73 (s, ^1^H), 8.67 (s, ^1^H), 8.11 (d, *J =* 5.0Hz, ^1^H), 7.52 (t, *J =* 6.0Hz, ^1^H), 7.24–7.31 (m, 3H), 6.10–6.17 (m, ^1^H), 3.05–3.25 (m, 2H), 3.02 (t, *J =* 7.5Hz, 2H), 1.95 (t, *J =* 7.0Hz, 2H), 1.65–1.70 (m, 2H), 1.46–1.50 (m, 4H), 1.44 (s, 9H), 1.24–1.28 (m, 4H), and 0.91 (t, *J =* 7.5Hz, 3H). HRMS (ESI) calcd for C_27_H_37_FN_6_O_4_S_2_ (M-H)^−^: 591.2229; found 591.2228.

##### 4.1.3.8 7-{[4-(2-(Tert-Butyl)-4-(2-Fluoro-3-(Phenylsulfonamido)Phenyl)Thiazol-5-yl)Pyrimidin-2-yl]Amino}-N-Hydroxyheptanamide (**21c**)

Yield: 75%. ^1^H NMR (400 MHz, DMSO-d_6_): *δ* (ppm) 10.30–10.34 (brs, 2H), 8.64 (s, ^1^H), 8.03 (d, *J =* 4.8Hz, ^1^H), 7.74 (d, *J =* 4.2Hz, ^1^H), 7.54–7.58 (m, ^1^H), 7.48–7.54 (m, 2H), 7.35–7.37 (m, ^1^H), 7.23 (t, *J =* 5.2Hz, ^1^H), 7.13–7.18 (m, 2H), 5.80–5.95 (brs, ^1^H), 3.08–3.20 (m, 2H), 1.94 (t, *J =* 7.2Hz, 2H), 1.44–1.52 (m, 4H), 1.40 (s, 9H), and 1.23–1.30 (m, 4H). HRMS (ESI) calcd for C_30_H_35_FN_6_O_4_S_2_ (M-H)^−^: 625.2072; found 625.2065.

##### 4.1.3.9 7-{[4-(4-(2-Fluoro-3-(Methylsulfonamido)Phenyl)-2-Methylthiazol-5-yl)Pyrimidin-2-yl]Amino}-N-Hydroxyheptanamide (**21d**)

Yield: 72%. ^1^H NMR (400 MHz, DMSO-d_6_): *δ* (ppm) 10.32 (s, ^1^H), 9.71 (s, ^1^H), 8.65 (s, ^1^H), 8.09 (d, *J =* 4.8Hz, ^1^H), 7.51 (t, *J =* 7.6Hz, ^1^H), 7.35–7.39 (m, ^1^H), 7.28–7.33 (m, ^1^H), 7.26 (t, *J =* 5.2Hz, ^1^H), 6.00-.620 (brs, ^1^H), 3.08–3.25 (m, 2H), 3.00 (s, 3H), 2.71 (s, 3H), 1.94 (t, *J =* 7.2Hz, 2H), 1.42–1.53 (m, 4H), and 1.20–1.33 (m, 4H). HRMS (ESI) calcd for C_22_H_27_FN_6_O_4_S_2_ (M-H)^−^: 521.1446; found 521.1440.

##### 4.1.3.10 7-{[4-(4-(3-(Ethylsulfonamido)-2-Fluorophenyl)-2-Methylthiazol-5-yl)Pyrimidin-2-yl]Amino}-N-Hydroxyheptanamide (**21e**)

Yield: 84%. ^1^H NMR (400 MHz, DMSO-d_6_): *δ* (ppm) 10.33 (s, ^1^H), 9.74 (s, ^1^H), 8.65 (s, ^1^H), 8.09 (d, *J =* 4.8Hz, ^1^H), 7.52 (t, *J =* 7.6Hz, ^1^H), 7.37 (t, *J =* 6.8Hz, ^1^H), 7.26–7.32 (m, 2H), 6.00–6.11 (brs, ^1^H), 3.10–3.20 (m, 2H), 3.05 (q, *J =* 7.2Hz, 2H), 2.71 (s, ^1^H), 1.94 (t, *J =* 7.2Hz, 2H), 1.48 (m, 4H), 1.26 (m, 4H), and 1.19 (t, *J =* 7.2Hz, 3H). HRMS (ESI) calcd for C_23_H_29_FN_6_O_4_S_2_ (M+H)^+^: 689.1748; found 537.1749.

##### 4.1.3.11 7-{[4-(4-(2-Fluoro-3-(Phenylsulfonamido)Phenyl)-2-Methylthiazol-5-yl)Pyrimidin-2-yl]Amino}-N-Hydroxyheptanamide (**21g**)

Yield: 80%. ^1^H NMR (500 MHz, DMSO-d_6_): *δ* (ppm) 10.26–10.35 (m, 2H), 8.65 (s, ^1^H), 8.02 (d, *J =*5.0 Hz, ^1^H), 7.75 (d, *J =* 5.0Hz, 2H), 7.61 (d, *J =* 7.5Hz, ^1^H), 7.51–7.55 (m, 2H), 7.39 (t, *J =* 7.5Hz, ^1^H), 7.20–7.27 (m, 3H), 5.75–5.90 (m, ^1^H), 3.05–3.20 (m, 2H), 2.67 (s, ^1^H), 1.94 (t, *J =* 7.5Hz, 2H), 1.42–1.52 (m, 4H), and 1.20–1.30 (m, 4H). HRMS (ESI) calcd for C_27_H_29_FN_6_O_4_S_2_ (M-H)^−^: 583.1603; found 583.1599.

### 4.2 Biological Study

#### 4.2.1 Cells and Agents

HCT116 and HT-29 cells were obtained from the Shanghai Cell Bank (Type Culture Collection (TCC), Chinese Academy of Sciences) and cultured in DMEM (10–013-CVR, corning) supplemented with 10% fetal bovine serum (04-001-1A, BI), 1% penicillin/streptomycin (03-031-1B, BI). Primary antibodies against ERK1/2 (4695s), phosphor-ERK1/2 (4370s), AKT (4685s), phosphor-AKT (13038, 4060), α-tubulin (2125S), acetyl-α-tubulin (5335), BRAF (9434), phosphor-BRAF (2696S), GAPDH 2118), and anti-rabbit or anti-mouse IgG horseradish peroxidase (HRP)–linked secondary antibodies were purchased from Cell Signaling Technology (Boston, MA, United States).

#### 4.2.2 Antiproliferation Cell Assay

The cells in the logarithmic phase were placed in 96-well plates (∼3000 cells/well) in a complete medium. After incubation overnight, the cells were exposed to the corresponding compounds or vehicle control at the indicated concentration for a further 72 h. Cell proliferation was evaluated using Cell Counting Kit 8 (CCK8, CK04, Dojindo Laboratories, Kumamoto, Japan). OD450 and OD650 were determined using a microplate reader. Absorbance rate (A) for each well was calculated as OD450 −OD650. The cell viability rate for each well was calculated as V% = (As − Ac)/(Ab − Ac) × 100%, and IC_50_ values were further calculated by concentration response curve fitting using GraphPad Prism 5.0 software. Each IC_50_ value is expressed as mean ± SD. As is the absorbance rate of the test compound well, Ac is the absorbance rate of the well without either the cell or test compound, and Ab is the absorbance rate of the well with the cell and vehicle control.

#### 4.2.3 *In Vitro* BRAF Enzymatic Activity Assay

BRAF^V600E^ (as BRAF^V599E^ in supplier’s catalog), BRAF (wild-type) and the Z′-Lyte Kinase Assay Kit were purchased from Invitrogen. The experiments were performed according to the instructions of the manufacturer. The final 10 μl reaction consists of 0.002 ng of BRAF, 10 ng of inactive MAP2K1 (MEK1), 100 ng of inactive MAPK1 (ERK2), 2 μM Ser/Thr3 peptide in 1×kinase buffer. For each assay, 10 μl kinase reactions were added to 384-well plate, mixed thoroughly, and incubated for 1 hour at room temperature. Then, a 5 μl development solution was added to each well and the plate was incubated for another 1 h at room temperature. Then, a 5 μl stop reagent was loaded to stop the reaction. For the control setting, a 5 μl phospho-peptide solution instead of kinase/peptide mixture was used as 100% phosphorylation control. Then, 2.5 μl 1.33×kinase buffer instead of ATP solution was used as 100% inhibition control, and 2.5 μl of 4% DMSO instead of compound solution was used as the 0% inhibitor control. The plate was measured on an EnVision Multilabel Reader (Perkin-Elmer). Curve fitting and data presentations were performed using GraphPad Prism, version 5.0. Every experiment was repeated at least two times.

#### 4.2.4 *In Vitro* HDAC Enzymatic Activity Assay

The purified recombinant Human HDACs and their corresponding substrates were purchased from BPS Bioscience (BPS Bioscience Inc., United States). The assays were carried out in a 384-well format using the BPS fluorescent–based HDAC activity assay according to the manufacturer’s protocol. In brief, 10 ml of the HDAC reaction mixture was composed of HDAC assay buffer, 100 mg BSA, serial diluted test compounds, appropriate concentration of HDACs, and 20 mM fluorogenic substrate, the mixture was incubated at 37°C for 60 min, and then stopped by the addition of developer containing trypsin and TSA. After 20 min of incubation, the fluorescence was detected at the excitation wavelength of 360 nm and the emission wavelength of 460 nm using the EnVision Multilabel Reader (PerkinElmer Inc., United States). The analytical software, GraphPad Prism 5.0 (GraphPad Software, Inc., United States) was used to generate IC_50_ value via non-linear regression analysis.

#### 4.2.5 Western Blot Analysis

The cells were treated with various concentrations of **14b** for 6 h. Then, the cells were lysed using 1×SDS sample lysis buffer (CST recommended) with protease and phosphatase inhibitors. Cell lysates were loaded and electrophoresed onto 8–12% SDS-PAGE gel, and then the separated proteins were transferred to a PVDF film. The film was blocked with 5% BSA (Sigma-Aldrich, St. Louis, MO, United States) in a TBS solution containing 0.5% Tween-20 for 4 h at room temperature, then incubated with the corresponding primary antibody (1:1000–1:200) overnight at 4°C. After washing with TBST, HRP-conjugated secondary antibody was incubated for 2 h. The protein signals were visualized by the ECL Western Blotting Detection Kit (Thermo Scientific, Grand Island, NY, United States), and detected with Amersham Imager 600 system (GE, Boston, MA, United States).

#### 4.2.6 *In Silico* Docking Study

The crystal structures of HDACs and BRAF ^V600E^ complexes (PDB IDs: 4BKX, 5EEI, and 4XV2) were used as receptors to predict the binding modes of **14b** using Autodock (Morris et al., 2009). Before docking, those protein structures were processed by removing water and adding hydrogens using Ambertools18 (Case et al., 2005). A single chain of all the protein structures was selected for docking. The protonation states of all amino acids and **14b** were determined using PROPKA3 (Olsson et al., 2011), followed by 10000 steps of energy minimization. The structure of **14b** was optimized at the B3LYP level of theory with the 6–31+g(d) basis set using Gaussian 16 (Frisch et al., 2016). Partial charges of the receptors and ligands for the docking study were calculated using AutodockTools following the Gasteiger’s method (Gasteiger and Marsili, 1980). The center of the native ligand was set as the grid box center with the grid spacing of 0.375 Å. The number of energy evaluations was set to 2500000. The Autodock4Zn zinc force field (Santos-Martins et al., 2014) was used in HDAC studies in addition to the Autodock standard force field to properly handle the metal zinc coordination.

## Data Availability

The raw data supporting the conclusion of this article will be made available by the authors, without undue reservation.
