# Supplementary material for: Discovery of BRAF/HDAC Dual Inhibitors Suppressing Proliferation of Human Colorectal Cancer Cells
Source: Front Chem. 2022 Jul 22;10:910353. doi: 10.3389/fchem.2022.910353 (PMC9354042; doi:10.3389/fchem.2022.910353)
Supplement: Supplementary file 1 [file DataSheet1.docx]

Supplementary Material

Discovery of BRAF/HDACs Dual Inhibitors Suppressing Proliferation of Human Colorectal Cancer Cells

Yingjun Li^1,†,*^, Yongjun Huang^2,†^, Huimin Cheng^3,†^, Xiuwen Yu^2^, Fang Xu^2^, Ruxi Qi^5^, Botao Dai^1^, Yujian Yang^1^, Zhengchao Tu^4^, Lijie Peng^2,^*, Zhang Zhang^2,^*

^1^Academy for Advanced Interdisciplinary Studies and Department of Chemistry, Southern University of Science and Technology, Shenzhen 518000, China

^2^International Cooperative Laboratory of Traditional Chinese Medicine Modernization and Innovative Drug Development of Chinese Ministry of Education (MOE), Guangzhou City Key Laboratory of Precision Chemical Drug Development, School of Pharmacy, Jinan University, Guangzhou, 510632, China

^3^XtalPi Inc, (Shenzhen Jingtai Technology Co., Ltd)., Shenzhen, 518100, China

^4^Guangzhou Institutes of Biomedicine and Health, Chinese Academy of Sciences, Guangzhou 510530, China

^5^Cryo-EM Center, Southern University of Science and Technology, Shenzhen 518055, China

*** Correspondence:**Corresponding Author: Yingjun Li (liyj@sustech.edu.cn); Zhang Zhang ([zzmoxue@163.com](mailto:zzmoxue@163.com)); Lijie Peng ([elva_0916@jnu.edu.cn](mailto:elva_0916@jnu.edu.cn))

† These authors contributed equally to this work.

**Table of Contents**：

Supplementary western blot analysis………………………………………….p2-p3

Enzymatic activity of vorinostat, **14j** and **21c** against HDAC8.…………….p3

NMR and HPLC spectra of compounds…………………………………….p3-p12

1. **
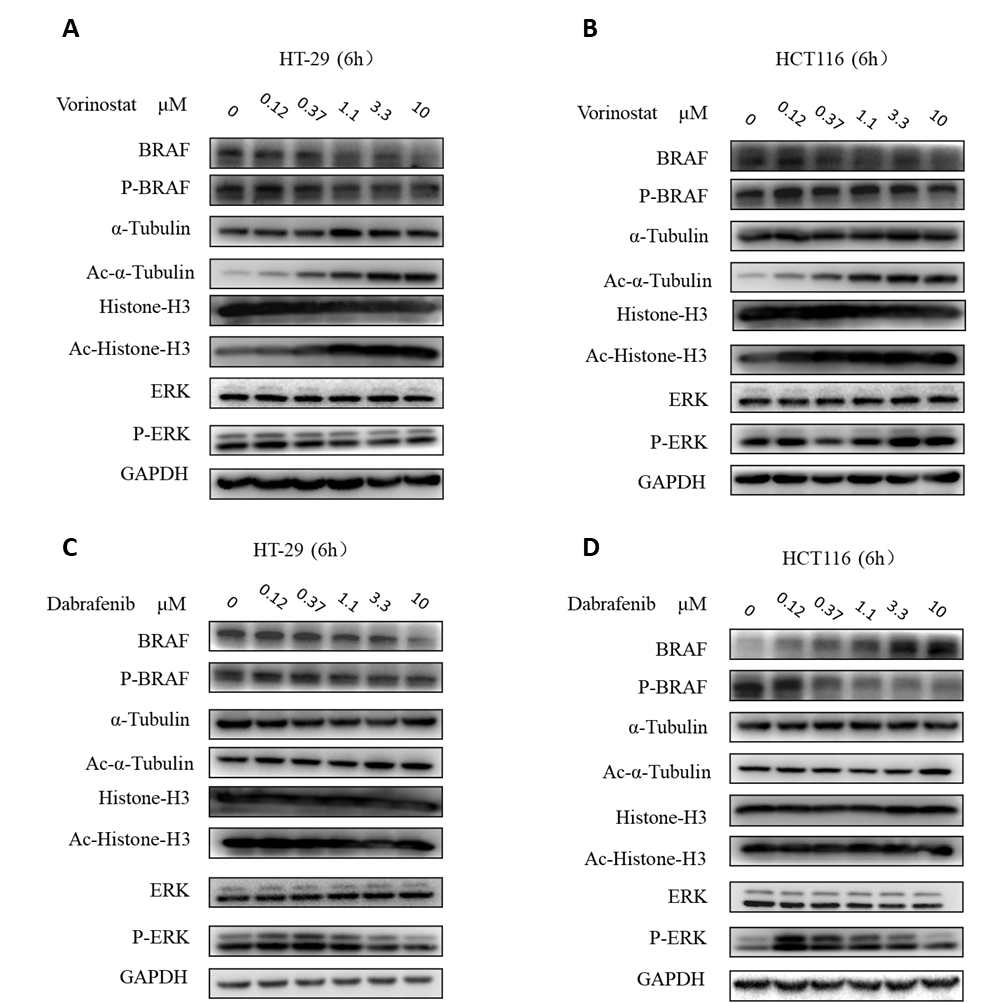
Supplementary Figures**

**Supplementary Figure 1.** The efforts of vorinostat and dabrafenib on the HDAC and RAF pathway in HT-29 and HCT116 cells. **A**. Vorinostat dose-dependently induced the acetyl-α-tubulin and acetyl-histone-H3 in HT-29 cells, but had little impact on the phosphorylation of ERK. **B**. Vorinostat dose-dependently induced the acetyl-α-tubulin and acetyl-histone-H3 in HCT116 cells, but had little impact on the phosphorylation of ERK. **C**. Dabrafenib reduced the phosphorylation of ERK, but had little impact on the acetyl-α-tubulin and acetyl-histone-H3 in HT-29 cells. **D**. Dabrafenib paradoxically induced the phosphorylation of ERK, and had little impact on the acetyl-α-tubulin and acetyl-histone-H3 in HCT116 cells.


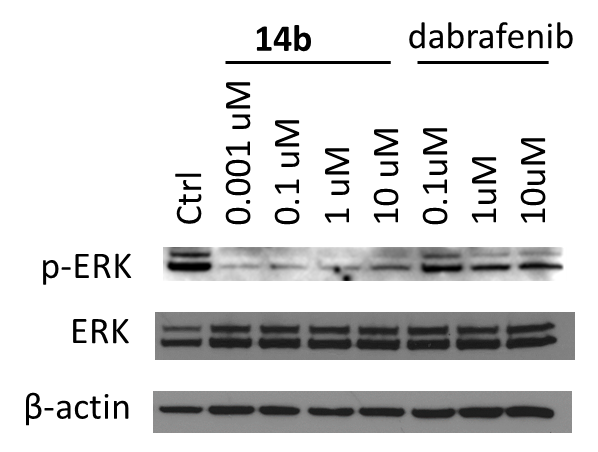


**Supplementary Figure 2.** **14b** inhibit the phosphorylation of ERK in B16 cells. B16 cells (mouse melanoma cells with wild type BRAF) were treated with **14b** or dabrafenib for 24 h, immunoblot analysis of P-ERK, Total-ERK and β-actin indicates **14b** downregulate P-ERK but dabrafenib does not.

**Supplementary Table 1.** Enzymatic Activity of Vorinostat, **14j** and **21c** against HDAC8.

| Cmpd | HDAC8 Enzymatic IC_50_ (μM) |
| --- | --- |
| Vorinostat | 1.158 |
| 14j | 0.876 |
| 21c | 0.623 |

# ABBREVIATIONS

HDAC, Histone Deacetylases; CRC, colorectal cancer; ERK, MAPK, mitogen-activated protein kinase; BRAFi, BRAF inhibitors; mCRC, metastatic colorectal cancer, EGFR, epidermal growth factor receptor; SAR, the structure-activity relationship; Ac-histone H3, acetylated histone 3; Ac-α-tubulin, acetylated α-tubulin; ERK, extracellular signal-regulated kinase; p-ERK, phosphorylated extracellular signal-regulated kinase.

# ^1^H NMR spectra, HRMS spectra and HPLC analysis of compounds

^1^H NMR spectra of compound **14a**

^1^H NMR spectra of compound **14b**

**
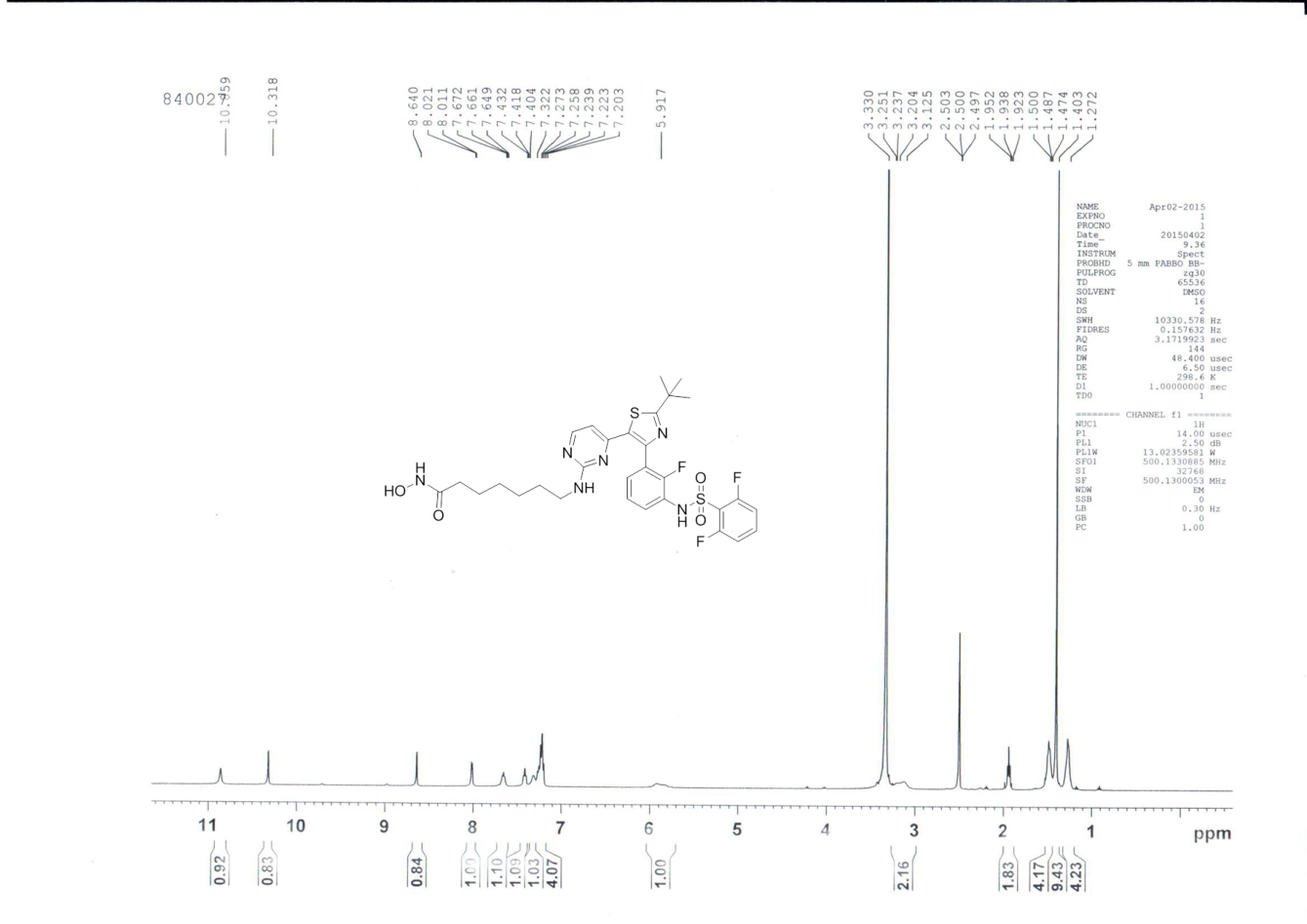
**

^1^H NMR spectra of compound **14c**

**
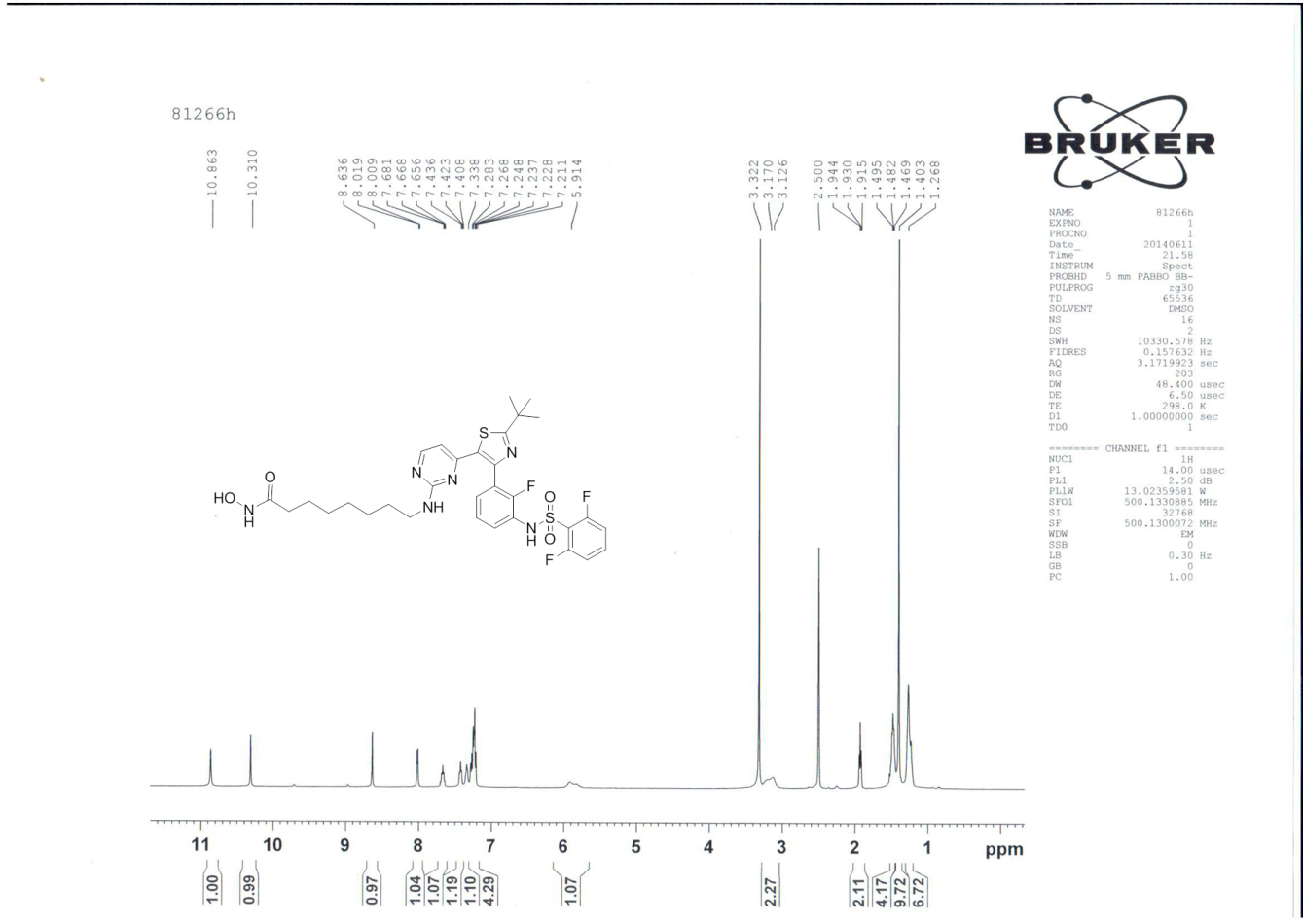
**

^1^H NMR spectra of compound **14d**

^1^H NMR spectra of compound **14e**

^1^H NMR spectra of compound **14f**

^1^H NMR spectra of compound **14g**

**
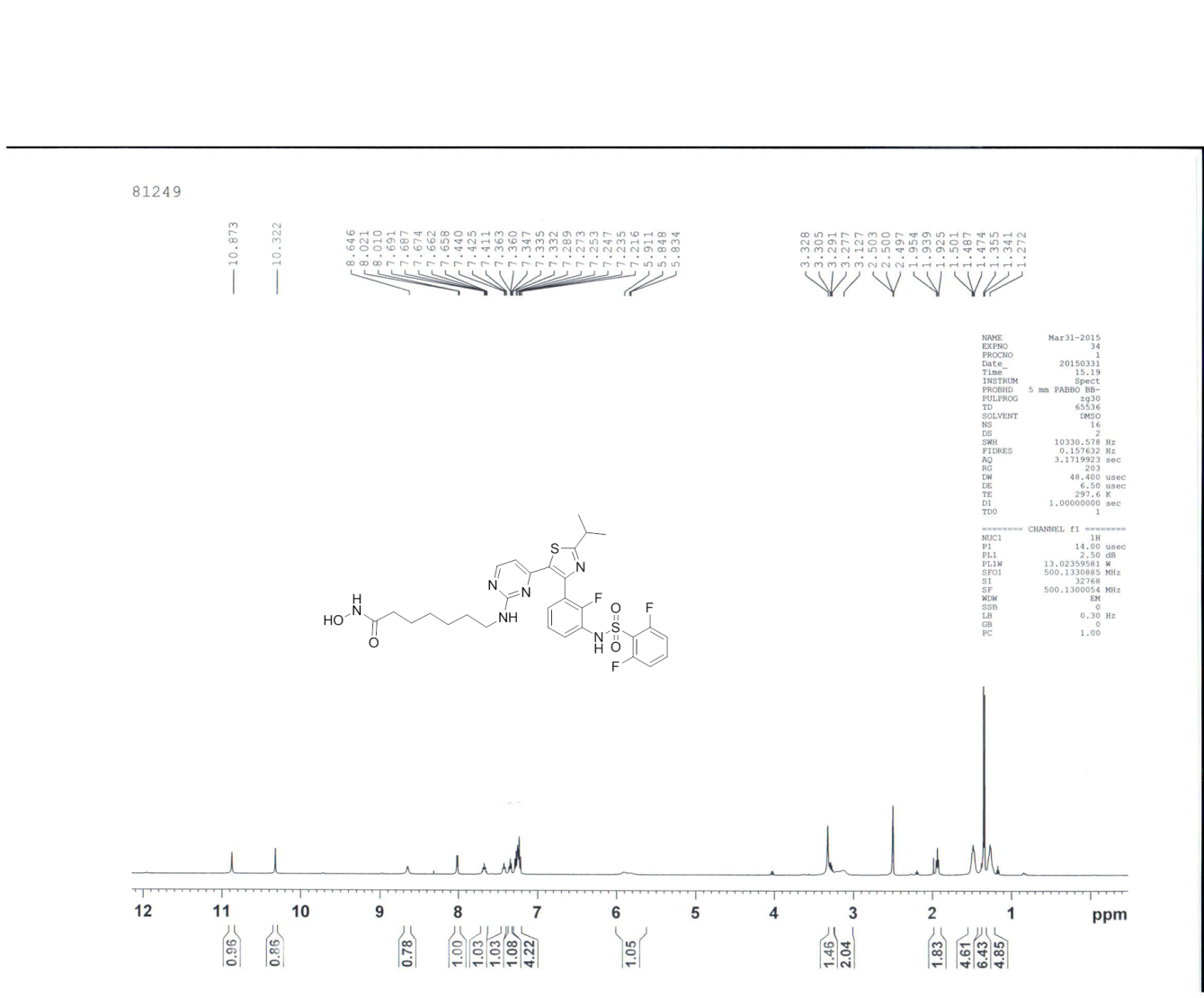
**

^1^H NMR spectra of compound **14h**

**
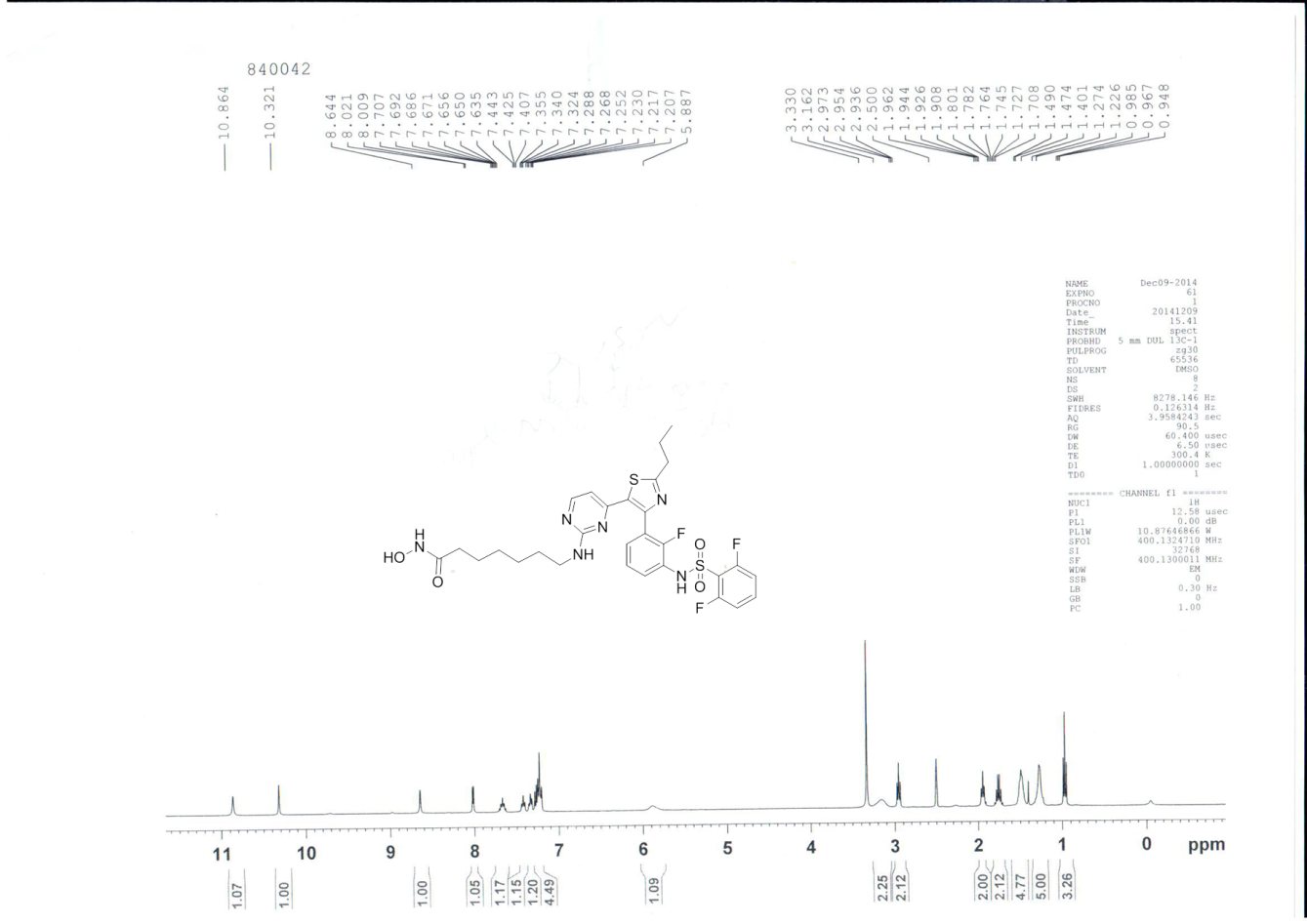
**

^1^H NMR spectra of compound **14i**

^1^H NMR spectra of compound **21a**

**
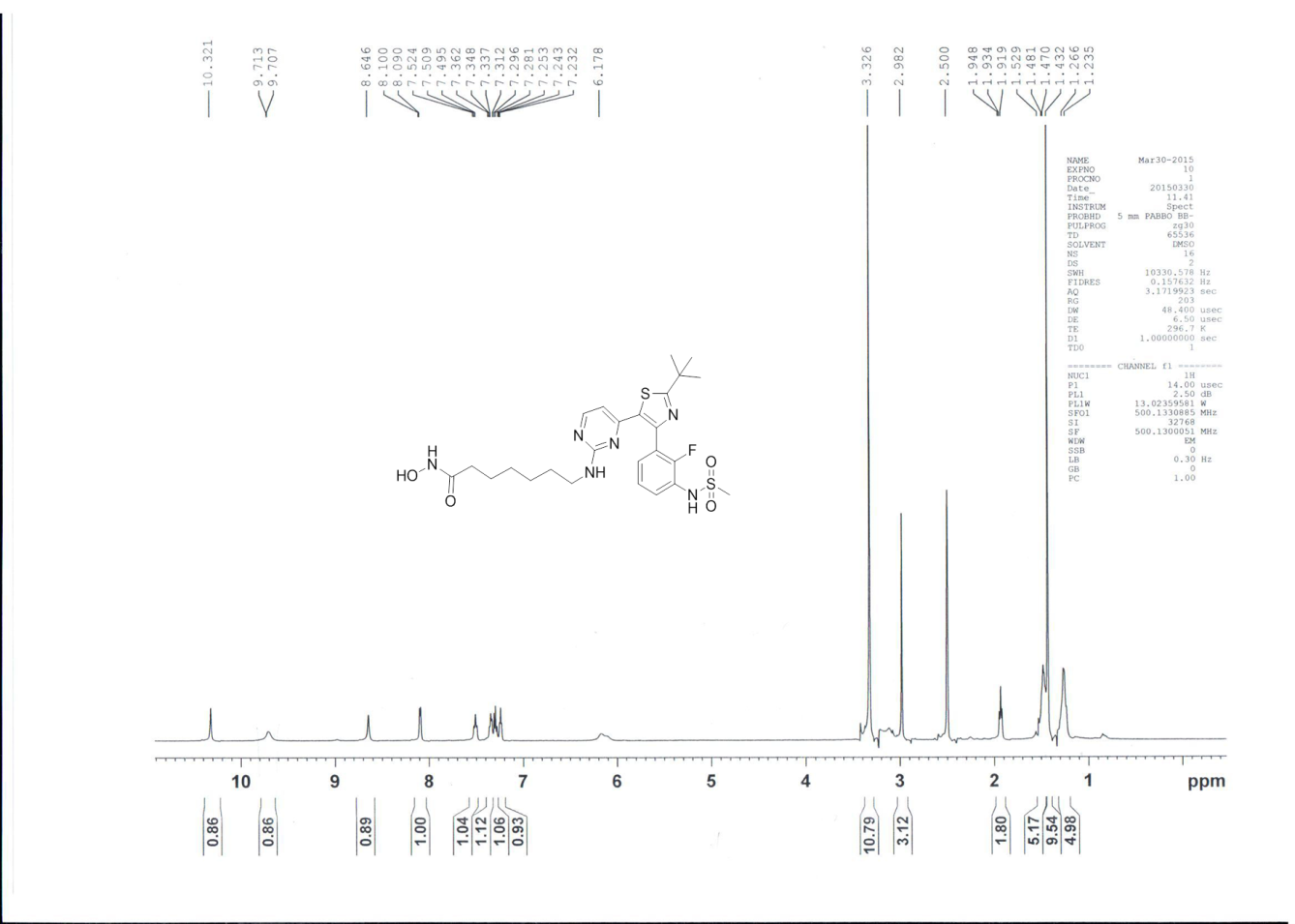
**

^1^H NMR spectra of compound **21c**

^1^H NMR spectra of compound **21d**

^1^H NMR spectra of compound **21e**

^1^H NMR spectra of compound **21f**

HPLC of **14b**


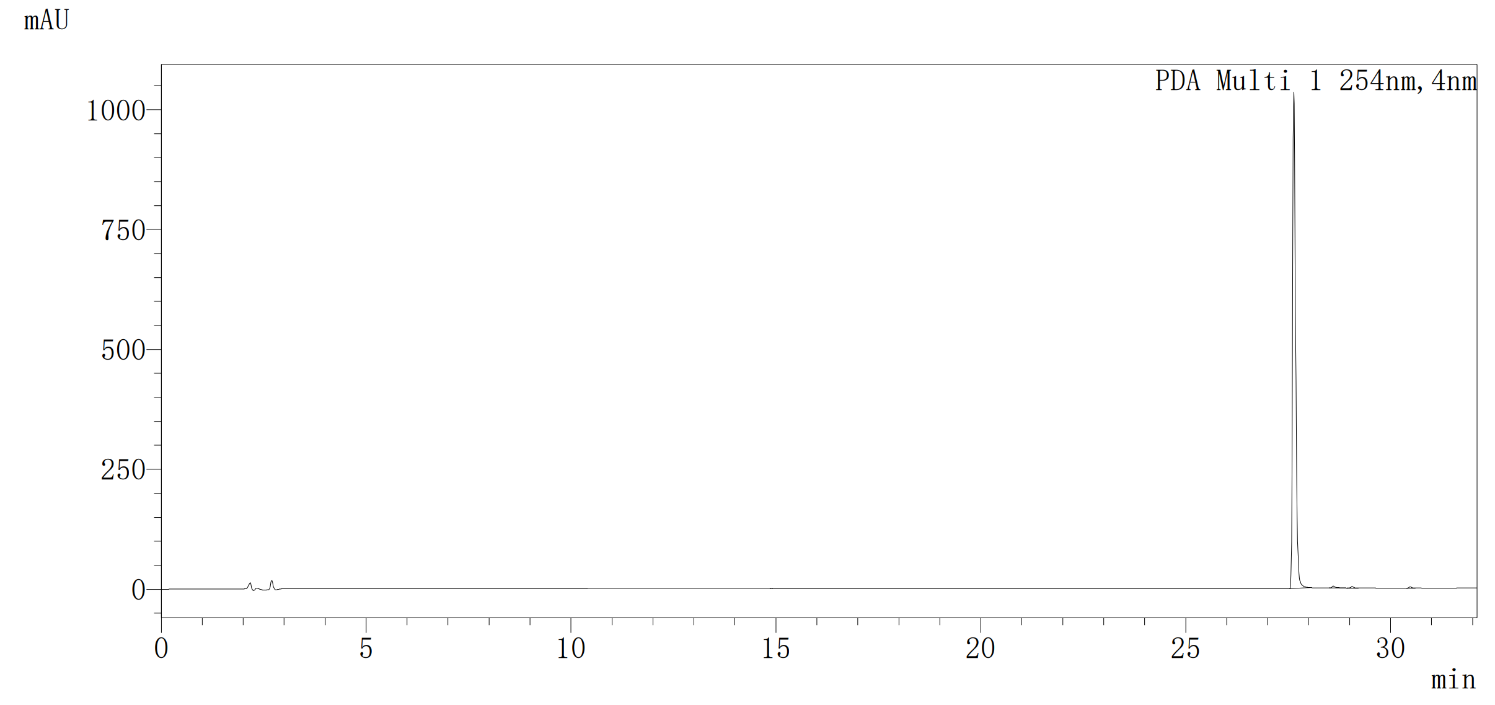


98.893
